# Therapeutic impact of BET inhibitor BI 894999 treatment: backtranslation from the clinic

**DOI:** 10.1038/s41416-022-01815-5

**Published:** 2022-04-20

**Authors:** Ulrike Tontsch-Grunt, Paula-Elena Traexler, Anke Baum, Hanny Musa, Kristell Marzin, Shaonan Wang, Francesca Trapani, Harald Engelhardt, Flavio Solca

**Affiliations:** 1 Boehringer Ingelheim RCV GmbH & Co KG, A-1120 Vienna, Austria; 2 Boehringer Ingelheim Pharma GmbH & Co. KG, Biberach, Germany; 3 Boehringer Ingelheim Pharma GmbH & Co. KG, Ingelheim am Rhein, Germany

**Keywords:** Drug development, Pharmacology

## Abstract

**Background:**

BET inhibitors have been tested in several clinical trials where, despite encouraging preclinical results, substantial clinical benefit in monotherapy remains limited. This work illustrates the translational challenges and reports new data around the novel BET inhibitor, BI 894999. At clinically achievable concentrations, mechanistic studies were carried out to study pathway modulation and rational drug combinations.

**Methods:**

BRD-NUT fusions are oncogenic drivers in NUT carcinoma (NC). The effects of BI 894999 on proliferation, chromatin binding and pathway modulation were studied in NC in vitro. These studies were complemented by efficacy studies either as a single agent or in combination with the clinical p300/CBP inhibitor CCS1477.

**Results:**

Based on the modelling of preclinical and clinical data, we proposed and implemented a new clinical scheduling regimen. This led to plasma levels sufficient to fully dislodge BRD-NUT from chromatin and to sustained and pronounced pharmacodynamic (PD) modulation of HEXIM1 and HIST2H2BF. Platelet counts in patient blood samples were improved compared to previous schedules. Rational combination studies of BI 894999 performed at clinically meaningful concentrations led to tumour regressions in all NC xenograft models tested.

**Conclusions:**

BI 894999 holds significant potential as a combination drug and CCS1477 p300/CBP inhibitor is a promising partner for future clinical trials.

## Background

**Fig. 1:**
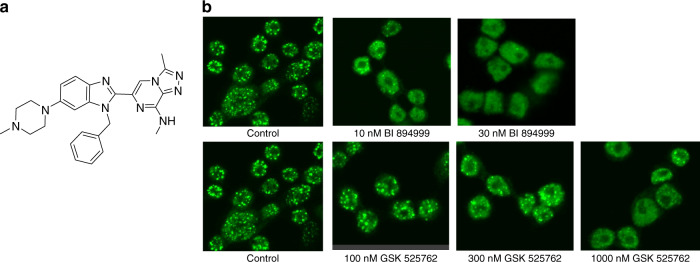
BI 894999 and the detaching of BRD-NUT from chromatin. **a** Structure of the oral BET inhibitor BI 894999. **b** Immunofluorescence staining of NUT megadomains on the NC cell line Ty-82 treated with BET inhibitors for 30 min. BRD4-NUT detaches from chromatin with 10–30 nM BI 894999 (speckled staining changes to fuzzy staining).

Bromodomain (BRD) proteins are chromatin ‘readers’; they bind to and interpret post-translational modifications, added by epigenetic ‘writers’ or removed by epigenetic ‘erasers’ and thus ultimately regulate gene expression. BRD4 is a member of the bromodomain and extra terminal domain protein (BET) family, consisting of four members BRD2, BRD3, BRD4 and BRDT. BRD4, like other BET family members, contains two bromodomains, BRD4-BD1 and BRD4-BD2 that bind to acetylated lysine residues on histone H3 and H4. BRD4 activates gene expression by activating P-TEFb (consisting of CDK9 and CyclinT1) which then can phosphorylate Ser2 on paused RNA Pol II, leading to transcriptional elongation [1–5]. Loss of BRD4 function, both by genetic means as well as by treatment with a BRD4 inhibitor, leads to sustained inhibition of proliferation of tumour cells, induction of differentiation, and ultimately apoptosis. BI 894999 is a novel triazolopyrazine small molecule oral BET inhibitor, synthesised through structure-based drug design efforts (WO2014076237; Fig. 1). BI 894999 is structurally distinct from BET inhibitors with a benzodiazepine scaffold, such as e.g. JQ1, OTX015, CPI-0610 or GSK525762. The preclinical efficacy and safety profile of BI 894999 supports its clinical evaluation in cancer patients. BI 894999 was tested in an open-label Phase I trial, which is now completed (NCT02516553).

NUT Carcinoma (NC) is a rare, extremely aggressive, poorly differentiated squamous cell carcinoma with a median overall survival of about 6.5 months. NC affects patients of all ages, from newborns to octogenarians, with a median age at diagnosis of 25 years. NC is defined by a genetic translocation involving the *NUTM1* gene and bromodomain-containing proteins, hence is an archetypal BET fusion-driven cancer [6–8].

BRD4-NUTM1 fusions occur in the majority (70%) of NC cases, with less frequent NUTM1 rearrangements involving either BRD3 or NSD3 (15% and 6% of cases, respectively) [9]. Diagnosis of NC is confirmed in tumour biopsies or surgical specimens based on the histologic finding of an undifferentiated or poorly differentiated squamous cell carcinoma and the immunohistochemical detection of the expression of the NUT protein [7, 10, 11]. Nowadays, the disease is more frequently diagnosed through the detection of the *NUTM1* gene rearrangements in panels of next-generation sequencing (NGS)-platforms. Apart from NC, no other genetic alterations affecting BRD4 have been described.

NC is an extremely aggressive disease where patients have few effective therapeutic options. The mechanistic role of BET proteins in the pathogenesis of the disease means that BET inhibitors could be a treatment alternative for NC patients (http://www.nmcregistry.org/clinictrial.html; http://www.clinicaltrials.gov). Indeed, NC patients have been included in clinical trials of several BET inhibitors. Although proof of concept was achieved, the overall clinical benefit has been disappointing [12–15]. Current BET inhibitors do not discriminate between the paralogs BRD4, BRD2 and BRD3, resulting in a narrow safety window, with thrombocytopenia as the main dose-limiting toxicity. Thrombocytopenia is linked to inhibition of BRD3, functionally required during megakaryocyte and erythroid cell maturation [16]. Appropriate clinical dosing schedules are required to mitigate potential adverse events.

P300 and CBP (CREB-binding protein) are histone acetyltransferases (HAT) paralogs that impact the expression of cancer-driving genes. CCS1477 is a p300/CBP inhibitor [17] that binds non-selectively to the bromodomain pocket of p300 and CBP. CCS1477 is currently being tested in clinical trials (NCT04068597, NCT03568656). BRD4-NUT is tethered to acetylated chromatin by BRD4 bromodomains and the NUT portion of the fusion protein recruits p300, leading to increased local histone acetylation and a feed-forward mechanism to further recruit BRD4-NUT [18, 19]. Combination with p300 inhibitors may deepen the activity of BET inhibitors in the NC setting. Although studies to combine these inhibitors, either with two separate compounds [20], or with a single molecule co-targeting both entities [21] have been reported, this has been restricted to non-clinical tool compounds. We now provide in vivo preclinical confirmatory data for two Phase I compounds. Providing tolerability concerns can be overcome, the combination of these agents could represent an effective therapeutic option for NUT carcinoma patients.

In our studies we provide data back-translating findings from the clinical trial with BI 894999 to identify concentrations that were achievable in the clinic. Using the compound at these concentrations, the displacement of BRD-NUT from chromatin was evaluated and connected to human pharmacokinetic and pathway modulation markers, such as HEXIM1 and HIST2H2BF. In addition, we investigated the combination with the most promising partner p300/CBP inhibitor in in vivo tumour models.

## Methods

### Compounds

BI 894999 (WO2014076237) is the clinical compound synthesised by Boehringer Ingelheim. GSK525762 (I-BET762) and CCS1477 were purchased from Chemie Tek, Indianapolis, USA; NEO2734, A485 and Romidepsin were purchased from SelleckChem.

### BRD4 AlphaLISA assay

This bead-based proximity assay is based on the competitive displacement of a histone peptide (acetyl-histone H4/Lys5, 8, 12, 16) from the human bromodomain [22].

### Cell lines and cell viability assay

The NUT Carcinoma cell line Ty-82 was purchased from JCRB Japan No. 1330 and cultivated in RPMI 1640 supplemented with 10% fetal calf serum (FCS). The NC cell lines 10–15, 14169 and 10326 were in-licensed from Christopher French, Brigham & Womens Hospital, Boston, and were cultivated in DMEM supplemented with glutamax and 10% FCS.

Viability assays were performed on 96-well flat-bottom microtiter plates and incubated with Alamar Blue solution (Invitrogen, Carlsbad, US-CA) [23]. The effects of drug combinations were analysed using the Bliss Independence Model [24].

### Incucyte assay

Live-cell imaging was performed with the Incucyte^®^ S3 Live-Cell Analysis System as described previously for Incucyte^®^ Zoom [22].

### Immunofluorescence staining of megadomains

Cells were seeded on four-well chamber slides (Merck Millipore, #PEZGS0416). After treatment, cells were fixed with 4% PFA (16% Solution, Thermo Fischer #28908 Paraformaldehyde, diluted 1:4 in PBS), permeabilised with 0,1% TX-100 (Sigma #93443, diluted in PBS). After blocking in blocking buffer (10% normal goat serum, Sigma #G9023–10ml, diluted in blocking solution: 2% BSA/PBS, Sigma #A7284–50ml, diluted 1:15 in PBS), the first antibody mAB rabbit anti-NUT 1:1000 (clone C52B1, Cell Signaling #3625) in blocking buffer was added, followed by incubation with the 2nd antibody goat anti-rabbit Alexa*488 1:1000 (Thermo Fisher, #A-11008) in blocking buffer in the dark. Cells were mounted with ProLong Gold Antifade Mountant with DAPI (Thermo Fisher, #P36931) and analysed using a Laser scanning microscope (LSM700).

### In vivo studies in mice

Mice were group-housed (8–10 mice per cage) under pathogen-free and controlled environmental conditions (21 ± 1.5 C temperature, 55 ± 10% humidity) in an AAALAC accredited facility and handled according to the institutional, governmental and European Union guidelines (Austrian Animal Protection Laws, GV-SOLAS and FELASA guidelines). Animal studies were approved by the internal ethics committee and the local government committee. Mice were 6- to 8-week-old female CIEA NOG purchased from Taconic, Denmark (NOD.Cg-*Prkdc*^*scid*^ *Il2rg*^*tm1Sug*^/JicTac). 5 × 10^6^ NC tumour cells were injected subcutaneously into the right flank of the mice (1 site per mouse). Mice were randomly distributed between the treatment and the vehicle control when tumours were well established and had reached volumes of 100–150 mm^3^. Compounds were suspended in 0.5% Natrosol and administered once daily intra-gastral by gavage needle. Tumour diameters were measured three times a week with a calliper. The volume of each tumour [in mm3] was calculated according to the formula “tumour volume = length × diameter^2^ × π/6.” The median of the tumour volume of each treatment group T was referred to as the median of the control C as tumour growth inhibition TGI) defined as: TGI = 100 × {1−[(treated _final day_ − treated _day1_) / (control _final day_ − control _day1_)]}. One-sided non-parametric Mann–Whitney–Wilcoxon U-tests were applied to compare all treatment groups with the control, as well as the combination therapy group with the corresponding monotherapy groups, looking for a decrease in the tumour volume (inhibition of tumour growth, efficacy parameter) and a change in body weight (body weight loss as tolerability parameter). Within each subtopic the *p*-values of the efficacy parameters were adjusted for multiple comparisons according to Bonferroni–Holm. The *p*-values of the tolerability parameter remained unadjusted in order not to overlook a possible adverse effect. The level of significance was fixed at α = 5%. An adjusted *p*-value of less than 0.05 was considered to show a statistically significant difference between the groups. The statistical evaluation was prepared using the software Graph Pad Prism.

### Histopathological analysis of NC xenografts tumours

Immunohistochemistry was performed on formalin-fixed, paraffin-embedded tumour tissue using antibodies against human Ki67(Cell Signaling #9027, 1:400 dilution), anti-human mitochondria (Abcam ab92824, 1:1000 dilution) and against endogenous NUT protein (Cell Signaling #3625, 1:50 dilution) used to detect NUT fusion proteins [7, 10]. Digital images of whole tissue sections were acquired using a Leica SCN400 histology scanner (Leica Microsystems). Ki67-positive index was evaluated using Halo Software V. 3.2 (Indica lab). Regions of interest (ROIs) within the tissue sections were first identified using Halo Software via machine learning technology across pathological samples and tissue control, within these ROIs, nuclei were detected and classified as positive or negative based on IHC staining thresholds.

### Clinical schedule

Schedule B (NCT02516553): daily oral treatment from Day 1 to Day 14 at 2.5 mg/day followed by a week-off in 3-week cycles. Schedule C (NCT02516553): one week-on, one week-off treatment, repeated every two weeks in 4-week cycles with, at each treatment week, a loading dose (6 mg) on Day 1 followed by a maintenance dose (3 mg per day) on the following 6 days.

### RNA isolation of NC cells and sequencing

RNA was isolated according to state-of-the-art protocols. In brief, cells were lysed in TRI lysis reagent (Sigma–Aldrich, T9424, St. Louis, US-MO). RNA-seq sequencing libraries were prepared using the QuantSeq 3’ mRNA-Seq Library Prep Kit FWD for Illumina kit (Lexogen, # 015.96) and subsequently sequenced on an Illumina NextSeq 500 system.

### Gene expression profiling (RNA-seq) and differential gene expression analysis

Gene expression profiling and differential gene expression analysis were performed as previously described [25]. Sequencing reads were processed with a pipeline, building upon the implementation of the ENCODE’ “Long RNA-seq” pipeline: reads were mapped against the Homo sapiens (human) genome hg38/GRCh38 (primary assembly, excluding alternate contigs) using the STAR (v2.5.2b) aligner allowing for soft clipping of adapter sequences. For quantification, we used transcript annotation files from Ensembl version 86, which corresponds to GENCODE 25. Samples were quantified with the above annotations, using RSEM (v1.3.0) and featureCount (v1.5.1). Quality controls were implemented using FastQC (v0.11.5), picardmetrics and dupRadar (v1.0.0) at the respective steps. Differential expression analysis was performed on the mapped counts derived from featureCount using limma/voom. If not otherwise stated, an absolute log2 fold change cut-off of 1 and a false discovery rate (FDR) of <0.05 was used for all analysis.

### RNA isolation and analysis

Total RNA was extracted from whole blood using PAXgene Blood RNA kit (Qiagen) following the manufacturers’ instructions. Total RNA was quantified by direct absorbance measurement at 260 nm using a NanoDrop Spectrophotometer. Quality check was performed by additional measurement of absorbance at 280 nm and calculation of the absorbance ratio OD260/OD280.

### Nanostring analysis

mRNA expression was assessed using a custom NanoString nCounter codeset generated for the genes of interest plus housekeeping genes. In brief, probes were hybridised to 150 ng of total RNA for 19 h at 65 °C and applied to the nCounter™ Preparation Station for automated removal of excess probe and immobilisation of probe‐transcript complexes on a streptavidin‐coated cartridge. Data was collected using the nCounter™ Digital Analyzer by counting the individual barcodes.

Data were analysed using an in-house analysis pipeline that follows the latest Nanostring Technologies standards for the analysis of nCounter data. The analysis pipeline includes QC of the raw data using the NACHO [26]. The housekeeping genes ACTB, GAPDH, HPRT1, MGAT1 and TMED9 were used for normalisation. After normalisation expression values were exported and used for figure generation using ggplot22 [27].

## Results

### BI 894999 is a potent BET inhibitor

BI 894999 is an acetyl-lysine mimetic that inhibits the binding of bromodomain BRD4-BD1 and BRD4-BD2 (IC_50_ of 5 ± 3 nM and 41 ± 30 nM, respectively) to acetylated histone (Lys5, 8, 12, 16).

The cellular potency of BI 894999 was previously determined in cell proliferation assays across large panels of human solid and haematological cancer lines as well as primary patient-derived AML samples [23, 28, 29]. BI 894999 was found to be at least 10-fold more potent when compared to other BET inhibitors (GSK525762, OTX015, JQ1). Here we expanded the scope of our evaluations to include four NC cell lines for the in vitro and in vivo experiments: Ty-82, 10–15, 14169, containing the BRD4-NUT fusion and 10326 containing the BRD3-NUT fusion. In cell proliferation assays, the GI_50_ values for BI 894999 in all NC lines were in the low single-digit nM range (geomean from 0.9 to 2.6 nM), thus ~40–60-fold more potent than JQ1 and GSK525762 and 10–20 more potent than the dual BET/p300 inhibitor NEO2734, depending on the NC cell line (Table 1B, Supplementary Figs. S1, S2).

**Table 1:** Sensitivity of BI 894999.

(A) Binding assay (AlphaLISA) for BRD4-BD1 and BRD4-BD2 for different BET inhibitors.				
Binding assay (AlphaLISA)	IC_50_ [nM]			
Bromodomain	BI 894999	GSK525762	OTX015	JQ1
BRD4-BD1	5 ± 3	262 ± 88	200 ± 119	111 ± 74
BRD4-BD2	41 ± 30	137 ± 93	153 ± 34	104 ± 64

(B) Cell proliferation assay (Alamar readout) on four NC cell lines with BET inhibitors BI 894999, GSK525762, JQ1, with the dual BET and p300/CBP inhibitor NEO2734 and the p300/CBP inhibitor CCS1477.				
	geomean GI_50_ [nM]			
Compound	Ty-82 BRD4-NUT	10–15 BRD4-NUT	14169 BRD4-NUT	10326 BRD3-NUT
BI 894999	2.6	1.7	0.9	1.2
GSK525762	154.3	113	55.0	43.6
JQ1	109.8	84.4	49.2	43.2
NEO2734	26.5	22.6	20.4	11.2
CCS1477	38.5	32.9	62.9	22.7

The effect of BET inhibition on cell viability was also assessed. Notably, in contrast to AML cell lines, which are comparably sensitive (GI_50_ values in the single-digit nM range [28] and readily underwent apoptosis upon BI 894999 treatment, NC lines displayed growth arrest, upregulated markers of differentiation or senescence [21, 30] and required >100-fold higher compound concentrations to undergo cell death (Supplementary Fig. S3).

### BI 894999 treatment leads to fast displacement of BRD-NUT from chromatin

The acetyl-lysine–bromodomain interaction is crucial for the oncogenic function of BRD-NUT. The binding of BRD-NUT to chromatin results in large, hyperacetylated nuclear foci, so-called megadomains, which can be visualised with an antibody against the NUT protein [6, 19] as a typical speckled pattern (Fig. 1b). Treatment with BET inhibitors re-distributes BRD4-NUT and BRD3-NUT to a faint diffuse staining pattern. (Fig. 1b); however, it cannot be fully determined to what degree it is entirely evicted from chromatin. BRD-NUT detaches from chromatin within 30 minutes of treatment with BI 894999, at concentrations ranging between 10–30 nM in the BRD4-NUT cell line Ty-82, and at 3–10 nM BRD3-NUT 10326 cells. In comparison, 1000 nM of GSK525762, and >100 nM of the dual BET/p300 inhibitor NEO2734 were necessary to re-distribute BRD4-NUT. Concentrations up to 1000 nM of the p300/CBP inhibitor CCS1477 had no effect (Fig. 1b and Supplementary Fig. S4A-E). Washout experiments showed that the speckled staining (BRD-NUT bound to chromatin) was quickly restored. Subsequent treatment with compound (1 h BI 894999, 23 h medium) again resulted in re-localisation (data not shown). To mimic a pharmacokinetic profile in even more detail, Ty-82 cells were treated with 30 nM for 1 h, followed by washout and treatment with 10 nM or 5 nM or 2.5 nM or control medium for 23 h. Some degree of restoration of BRD-NUT chromatin binding could be visualised at 30 nM → 10 nM and 30 nM → 5 nM conditions, but less than after the switch to 2.5 nM. (Supplementary Fig. S5). These data were very informative to guide the identification of the required plasma levels in clinical trials.

### In vitro analyses and modelling guided the clinical schedule

We and others have previously identified and described the BET pathway modulation markers HEXIM1 [28, 31, 32] and HIST2H2BF, and have routinely used them throughout the clinical trial [33].

**Fig. 2:**
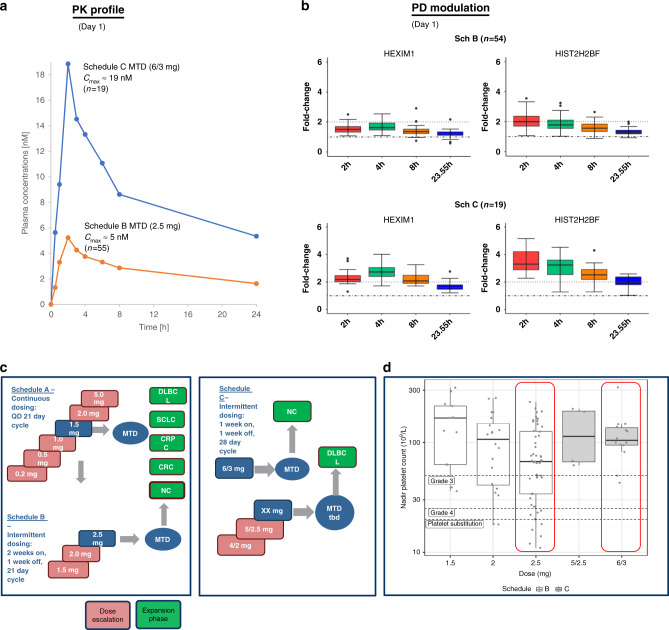
PK-PD analysis of BI 894999 in patient samples. **a** Day 1 BI 894999 plasma concentration-time profiles from patients treated under Sch B (orange line) or Sch C (blue line). Mean C_max_ of 5 nM for 55 patients in Sch B (MTD at 2.5 mg) and of 19 nM for 19 patients in Sch C (MTD at 6/3 mg) are displayed. **b** Nanostring analysis of pathway modulation markers HEXIM1 and HIST2H2BF. Fold change 2 h, 4 h, 8 h and 23.5 h after Day 1 treatment are plotted. **c** Details for clinical schedules. MTD was determined for NC, but not yet for DLBCL. **d** Measurement of platelet counts comparing Sch B and Sch C at different doses including MTD. Better tolerability at an increasing cumulative dose (over 6 weeks) in Sch C (72 mg) compared to Sch B (70 mg). Red boxes indicate the Nadir platelet counts at the MTD levels for Sch B (2.5 mg) and Sch C (6/3 mg).

In the clinical trial (NCT02516553), 55 patients were treated daily with 2.5 mg BI 894999 for 2 weeks, followed by a weeklong drug holiday over a 3-week cycle (referred to as Schedule B). Drug plasma exposure assessments in the patients demonstrated a maximum plasma concentration of 5 nM, which was accompanied by a distinct, albeit moderate (max. two-fold), pharmacodynamic modulation of the two genes, HEXIM1 and HIST2H2BF (Fig. 2b).

Based on the preclinical observations (immunofluorescence staining of the megadomains), this level of exposure is predicted to be insufficient to achieve effective displacement of BRD3 and BRD4 fusion proteins from chromatin. The main on-target side effect (thrombocytopenia) limits the possibility of simply increasing the dose. Therefore, PK/PD modelling studies were performed (Supplementary Fig. S6) to define dose/schedules that optimise target coverage. These alternative schedules have been transitioned to clinical trials and implemented as Phase I, Schedule C, consisting of a one week-on, one week-off treatment, repeated every two weeks in 4-week cycles. At each treatment week, a loading dose on Day 1 is given, followed by a maintenance dose on the following 6 days. The MTD (maximally tolerated dose) in patients was defined as 6 mg loading dose, 3 mg maintenance dose for this schedule. This resulted in a maximal plasma concentration (mean C_max_) of 19 nM in patients, that according to the preclinical megadomain re-distribution experiments would result in a clinically meaningful target coverage (Fig. 2a). Accordingly, PD modulation for both BETi genes was clearly more pronounced (up to four-fold) and prolonged, with two-fold change being still measurable 23.5 h after drug application (Fig. 2b). Nadir platelet counts from patients were analysed by dose and schedule. In patients, better tolerability was achieved with Schedule C, as measured by platelet nadir counts (Fig. 2d), while the 6-week cumulative dose (Schedule C (72 mg) was comparable to Schedule B (70 mg), when used at MTD levels. As anticipated from the modelling studies, target engagement through the loading dose design (Fig. 2b, Supplementary Fig. S6) was higher in Schedule C.

### Combination of BI 894999 with p300/CBP inhibitor shows strong in vitro efficacy and pathway modulation

**Fig. 3:**
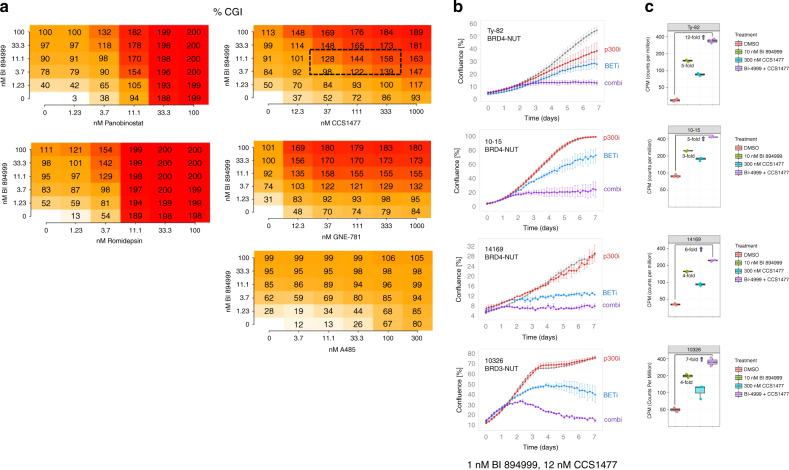
In vitro combination of BI 894999: proliferation and pathway modulation. **a** BI 894999 combined with various partners (HDACi Panobinostat and romidepsin, p300/CBPi CCS1477, GNE-781 and A485) in NC cell line Ty-82 and analysed for Bliss synergy. Combination platemap in a 6 × 6 matrix (duplicates for each setting) at the indicated concentrations. CGI (cell growth inhibition) Values <100%: tumour cell growth, CGI 100%: tumour cell stasis and GCI > 100%: tumour cell killing. **b** Incucyte life cell imaging analysis: BI 894999 combined with CCS1477 in four NC cell lines. Graphs are shown for 1 nM BI 894999 and 12 nM CCS1477 over 7 days of continuous treatment. **c** HEXIM1 expression analysis by RNA-seq in 4 NC cell lines. Cells were treated for 4 h with DMSO control, 10 nM BI 894999, 300 nM CCS1477 or a combination of both. Expression values in CPM (counts per million). Increased induction of HEXIM1 upon combination.

The effect of combining BI 894999 with different compounds on the proliferation of the NUT carcinoma cell line Ty-82 was analysed using the Bliss method [24]. After a larger screen, we focused further studies on HDAC and p300/CBP inhibitors, where the strongest synergy was observed (Fig. 3a). To be meaningful for clinical studies, cell growth inhibition >100% (indicative of cells undergoing cell death) must be reached at clinically achievable concentrations (10–20 nM for BI 894999). This was accomplished in combination with the HDAC inhibitors romidepsin and Panobinostat, as well as with the bromodomain-binding p300/CBP inhibitors GNE-781 and CCS1477, but not with the HAT domain binding p300/CBP inhibitor A485.

Combination with p300/CBP inhibitors, in particular the clinical compound CCS1477, was investigated the effect by Incucyte^®^ S3 live-cell imaging in four different NUT carcinoma cell lines (Fig. 3b). In all cases, a very strong combinatorial effect was seen, leading to full tumour cell growth inhibition or tumour cell death at doses of BI 894999 (1.3 nM) that, based on Phase I data, could be easily achievable in patients (Fig. 2a).

RNA-seq analysis after 4 h of in vitro treatment, demonstrated that pathway modulation (HEXIM1) was strongly increased upon the combination of BI 894999 (10 nM) and CCS1477 (300 nM) vs mono-treatment in all four NUT carcinoma cell lines (Fig. 3c). Although additional downmodulation of MYC was observed upon combination (especially in the 1015 cell line), these studies supported the view that a decrease in MYC levels is not a stable pathway modulation marker [28]. Basal levels of HIST2H2BF were very low in the four NC cell lines and therefore not suitable for additional analyses (Supplementary Figs. S7, 8).

### Combination of BI 894999 at clinically relevant doses with CCS1477 leads to tumour regression in three NUT carcinoma models

The lack of tolerability observed upon the combination of BI 894999 with HDAC inhibitors in mice limited the potential to evaluate this combination for further clinical development (data not shown; 2 mg/kg romidepsin applied q7d had to be reduced to 1 mg/kg due to body weight loss and did not show benefit when added to BI 894999 at clinically relevant doses).

BI 894999, CCS1477 or the combination of both were administered p.o. daily in three different NUT carcinoma xenograft models, two with BRD4-NUT fusion and one with BRD3-NUT fusion. CCS1477 was dosed at either 10 mg/kg or 5 mg/kg. BI 894999 was applied at 2 mg/kg, resulting in C_max_ in mice of 10 nM and in a ~2-fold HEXIM1 modulation in the blood (Supplementary Fig. S9)

**Fig. 4:**
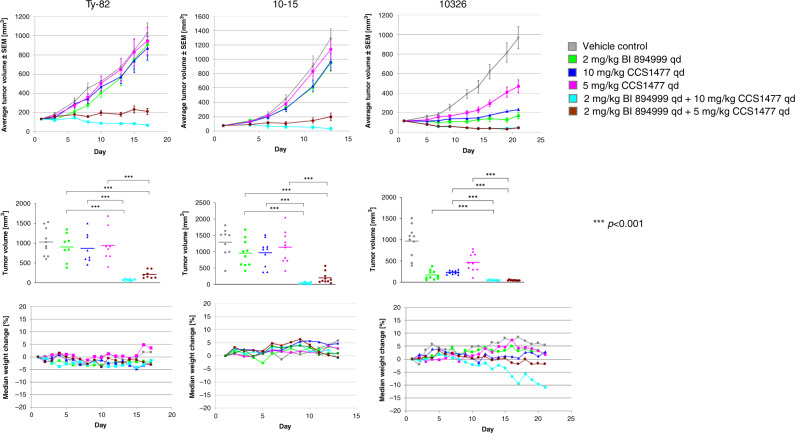
In vivo combination of BI 894999 with CCS1477. Upper row: Average tumour volumes (*n* = 10) ±SEM of three NC xenograft models (Ty-82: BRD4-NUT; 10–15: BRD4-NUT; 10326: BRD3-NUT). Middle row: Tumour volume of single mice (and average) at the end of the experiment. Lower row: Median body weight changes. Grey: vehicle control; green: 2 mg/kg BI 894999qd; blue: 10 mg/kg CCS1477 qd; magenta: 10 mg/kg CCS1477 qd; turquoise: combi 2 mg/kg BI 894999 and 10 mg/kg CCS1477; brown: combi 2 mg/kg BI 894999 and 5 mg/kg CCS1477 qd. One-sided non-parametric Mann–Whitney–Wilcoxon U-tests were applied to compare all treatment groups with the control, as well as the combination therapy group with the corresponding monotherapy groups. *p*-values were adjusted for multiple comparisons according to Bonferroni–Holm (details see Supplement Fig. 8).

Either agent applied as monotherapy had only weak effects on tumour growth, in the Ty-82 xenograft model, monotherapy treatment with either 2 mg/kg BI 89499, 5 or 10 mg/kg CCS1477 resulted in 5, 29 or 11% TGI (tumour growth inhibition), respectively, (Fig. 4, Supplementary Fig. S10). In contrast, the combination of both agents led to regressions. At the higher dose of 10 mg/kg for CCS1477, in combination with 2 mg/kg BI 89499, all tumours underwent regression (8/8 tumours, TGI of 107%), whereas at the lower dose of 5 mg/kg CCS1477, the combination of both drugs induced regression in 2/8 tumours, with an overall TGI of 94%.

Similarly, in the 10–15 xenograft model, TGI values of 40%, 24% and 30% for 2 mg/kg BI 894999, 5 mg/kg and 10 mg/kg, respectively, of CCS1477 were observed. In combination with 2 mg/kg BI 89499, combination with CCS1477 resulted in a TGI of 97% (2/10 tumours undergoing regression) and 103% (all mice showing tumour regressions) at 5 and 10 mg/kg, respectively.

The BRD3-NUT fusion xenograft 10326 xenograft was more sensitive, also in monotherapy. TGI values of 99%, 65% and 88% for 2 mg/kg BI 894999, 5 mg/kg and 10 mg/kg of CCS1477 were observed, respectively. In the combination studies, co-administration of 2 mg/kg and 10 mg/kg CCS1477 resulted in TGI of 108% and 107% respectively, with all tumours undergoing regression (Fig. 4). Notably, body weight loss was only observed in this model and at the highest combined dose of CCS1477 (2 mg/kg B I894999/10 mg/kg CCS1477). Analysis of drug plasma exposure after treatment confirmed no apparent drug–drug interaction in mice (Supplementary Fig. S11).

### Histopathological analysis of tumours derived from one NC model

**Fig. 5:**
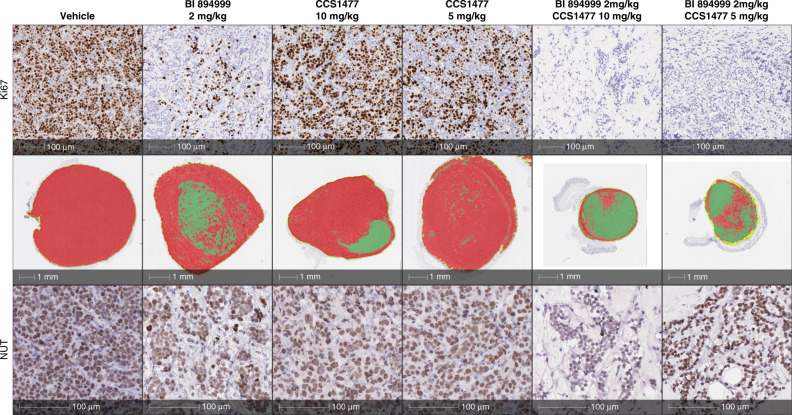
Histopathological analysis. BRD3-NUT 10326 tumour sections after treatment with 2 mg/kg BI 894999, 5 mg/kg or 10 mg/kg CCS1477 and the combination of both. Upper row: IHC staining for proliferation marker Ki67. Middle row: Pseudocolour staining based on human mitrochondria stain. Red = tumour, green = stroma. Lower row: IHC staining for NUT protein. Strong reduction of tumour viable area and complete absence of proliferating tumour cells with combination treatment. Effect of BET monotherapy on tumour cells may be underestimated by in vivo tumour measurement.

At the end of treatment of the NC 10326 xenograft experiment, on day 21, tumours from three mice from the control group and from each of the monotherapy groups and all tumours from the combination treatment groups were analysed by IHC. As assessed by Ki67 levels, BI 894999 monotherapy resulted in reduced cell proliferation, which was further decreased in the combination groups, being no longer detectable in the 2 mg/kg BI 894999/ 10 mg/kg CCS1477 combination setting (Fig. 5, Supplementary Figs. S12, S13). 2 mg/kg BI 894999 was sufficient to result in a complete loss of the speckled “megadomain” localisation, upon staining with the NUT-specific antibody, indicative of re-distribution of the BRD3-NUT fusion protein from chromatin. The effect was even more pronounced in the combination setting, but not seen following treatment with CCS1477 at either dose. The effect on the tumour architecture, morphological differentiation of the tumour cells and the infiltration of stromal cells was already visible in the NUT-stained sections (Fig. 5 lowest row and Supplementary Fig. S13), but more clearly seen after pseudocolour labelling, based on anti-human mitochondria staining. Only a ring of tumour cells was left after combination treatment, while the remainder of the tumour was filled with stromal components (Fig. 5 middle row). This might lead to an underestimation of the effectiveness of the combination treatment as assessed using calliper tumour measurement as a parameter.

## Discussion

No BRD inhibitor has been granted FDA approval to date and many clinical companies have stopped their development efforts (https://clinicaltrials.gov). Although there is a wealth of publications showing efficacy in cell lines and in preclinical in vivo models, either as monotherapy or in various combinations (reviewed by [34]), clinical experience with BET inhibitors has been fairly sobering and long-lasting responses scarce [12–15, 35–37]. One central issue is the narrow therapeutic window, in particular due to on-target adverse events, such as thrombocytopenia. This is considered to be largely due to BET proteins acting as general transcriptional regulators and the lack of selectivity of tested BET inhibitors across the different BET family members BRD2, BRD3 and BRD4. Notably, these adverse events may be underestimated in immune-compromised strains of mice used for preclinical testing, where relevant haemopoietic blood lineages are missing and that appear to intrinsically tolerate much higher doses of BETi (murine MTD concepts).

The short-lived antitumour response in solid cancer patients (including NC) may also be due to the emergence of resistance mechanisms [38]. An additional clinical benefit may therefore be gained by optimised dosing schedules together with the best possible rationale combinations for rapid and deep cell death in patients’ tumours. This may be even more clinically relevant in solid tumours where BET inhibitor treatment on its own results in growth arrest and induction of differentiation rather than cell killing [6, 39].

There is a pressing need to strengthen in-depth preclinical and translational research and back-translate findings from the bedside to the bench [40, 41]. Here we provide rationale and data for an optimised dosing schedule and a combination concept with clinical-stage compounds. These studies have focused on NC. Conceptually, NC is the most amenable tumour indication for BET inhibitors, as the characteristic BRD-NUT fusion has been shown to act as bonafide oncogenic drivers and thus provide an intrinsic and possible predictive biomarker for patient selection, a challenge in the clinical development of BETi [42].

Biochemical binding assays and cell proliferation assays demonstrated the excellent potency of BI 894999. In our view, the most informative assay was immunofluorescence staining of megadomains, using a NUT-specific antibody, which permits visualisation of re-distribution of BRD-NUT complexes from chromatin. When BRD-NUT is bound to chromatin, large regions can be observed as a speckled pattern of clear bright foci. Compound treatment results in re-distribution of the signal to a diffuse nuclear fluorescence signal. This assay permits, in a fast and simple way, visualisation of the inhibitory activity of BET inhibitors and estimation of the concentrations necessary for this effect. This assay was combined with PK-PD modelling to identify alternative dosing regimens (schedule C) that permit maximal plasma concentrations sufficient to ensure target coverage (about 20 nM in patients). Although this assay type did not allow us to determine if BRD-NUT detaches fully or only partially from chromatin, these predicted plasma levels for eviction were accompanied by sustained and prolonged pathway marker modulation. As predicted from the modelling studies, Schedule C resulted in less >Grade 3 thrombocytopenia events in patient than Schedule B at an equivalent 6-week cumulative dose.

Although initial tumour response was observed in some patients upon monotherapy with a BET inhibitor (ours and competitors), combination therapies are deemed necessary to attain durable responses [34, 42–46]. We focused on a combination partner with a strong mechanistic rationale for synergistic activity and the additional benefit of being clinically available. Data from French and colleagues showed that the NUT domain of chromatin-bound BRD-NUT fusion protein recruits the histone acetyl transferase (HAT) p300/CBP, leading to increased histone acetylation and the establishment of a feed-forward system that actively promotes transcription [8, 19].

As single agents, both BETi or p300/CBPi induced a deeper response in the 10326 BRD3-NUT fusion model, as compared to Ty-82 and 10–15 BRD4-NUT fusion models. This data is in line with a published analysis of 124 patients where patients with BRD-NUT3 or NSD3-NUT-positive fusion tumours were shown to have a better prognosis than patients with nonthoracic or thoracic primary tumours with BRD4-NUT [9].

Blocking both, p300/CBP and BET is a strategy that has already been considered by others using preclinical stage compounds [20, 21]. Published data with the dual BET-p300 inhibitor NEO2734 did not describe tumour regression but greater growth inhibition and significantly improved survival in mice bearing 10–15 xenografts, as compared to BETi alone [21]. In contradiction to Zhang et al. [20], our data suggest that only bromodomain-binding P300/CBP inhibitors, but not the HAT domain binding inhibitor P300/CBP inhibitor A485, show benefit in combination with a BET inhibitor. We concentrated on providing in vitro and in vivo efficacy data and PK/PD information for two Phase I compounds.

BI 894999 clinical PK, PD and tolerability data is available for the BET inhibitor whereas, for the p300/CBP inhibitor CCS1477 we relied on published information—one patient was administered with 50 mg/kg CCS1477 BD on a 3-day-on and 4-day-off schedule and one patient was treated with 25 mg/kg CCS1477 BD continuously [17]. Based on the described in vitro combination data and mouse xenograft studies, we defined a plasma concentration of 200–300 nM at 6 h after compound application to be sufficient for a synergistic effect (tumour regressions in the combination setting). This exposure could be achieved upon administration of 5 mg/kg CCS1477 in mice. As a single agent, this dose of CCS1477 did not result in efficacy either across the NC models tested here, as well as in published references [17]. Plasma exposure and potential dose-limiting toxicities remain to be reported for CCS1477 in patients.

Our data is encouraging and suggests that a combination of these clinical-grade compounds applied orally at low, tolerated doses, could represent a therapeutic option for NC tumour patients, while allowing management of potential adverse events. The first promising results from prostate cancer cells (Supplementary Fig. S14) suggest that this combination could also be applied beyond NC. This is in line with a recent report identifying EP300 (p300) and CREBBP (CBP) as a top hit synthetic lethal targets in a CRISPR screen upon BETi treatment of TNBC cell lines [47]. Broadening the above-described therapeutic combination concept in additional tumour indications warrants additional in vitro and in vivo studies. Clearly, the studies would benefit from the identification of a patient selection marker to enable and speed up clinical development in cancer patients.

## Conclusion

Our study provides preclinical in vitro and in vivo efficacy data for the oral BET inhibitor BI 894999. Pathway modulation analyses and studies on the binding and release of BRD/NUT from chromatin have focussed on the BET fusion-driven NUT carcinoma. Based on modelling of these interlinked preclinical data and clinical findings from Phase Ib (PK and PD modulation) we proposed and applied a clinical schedule offering the best balance of safety and efficacy.

This study extends knowledge of BET inhibitor BI 894999 as a combination drug and identified the p300/CBP inhibitor CCS1477 as a relevant combination partner in NUT carcinoma and supports further exploration in clinical trials.

## Supplementary information

Figures Supplement

Figure Legend Supplement

Reproducibility checklist

Form for open access

## Data Availability

The data generated in this study are available within the article and its supplementary data files. Data were also deposited in a repository: The data generated in this study are publicly available in Gene Expression Omnibus (GEO) at GSE183214.
