# Supplementary material for: Therapeutic impact of BET inhibitor BI 894999 treatment: backtranslation from the clinic
Source: Br J Cancer. 2022 Apr 20;127(3):577–86. doi: 10.1038/s41416-022-01815-5 (PMC9346113; doi:10.1038/s41416-022-01815-5)
Supplement: Supplementary file 1 — Figures Supplement [file 41416_2022_1815_MOESM1_ESM.pptx]

## Slide 1
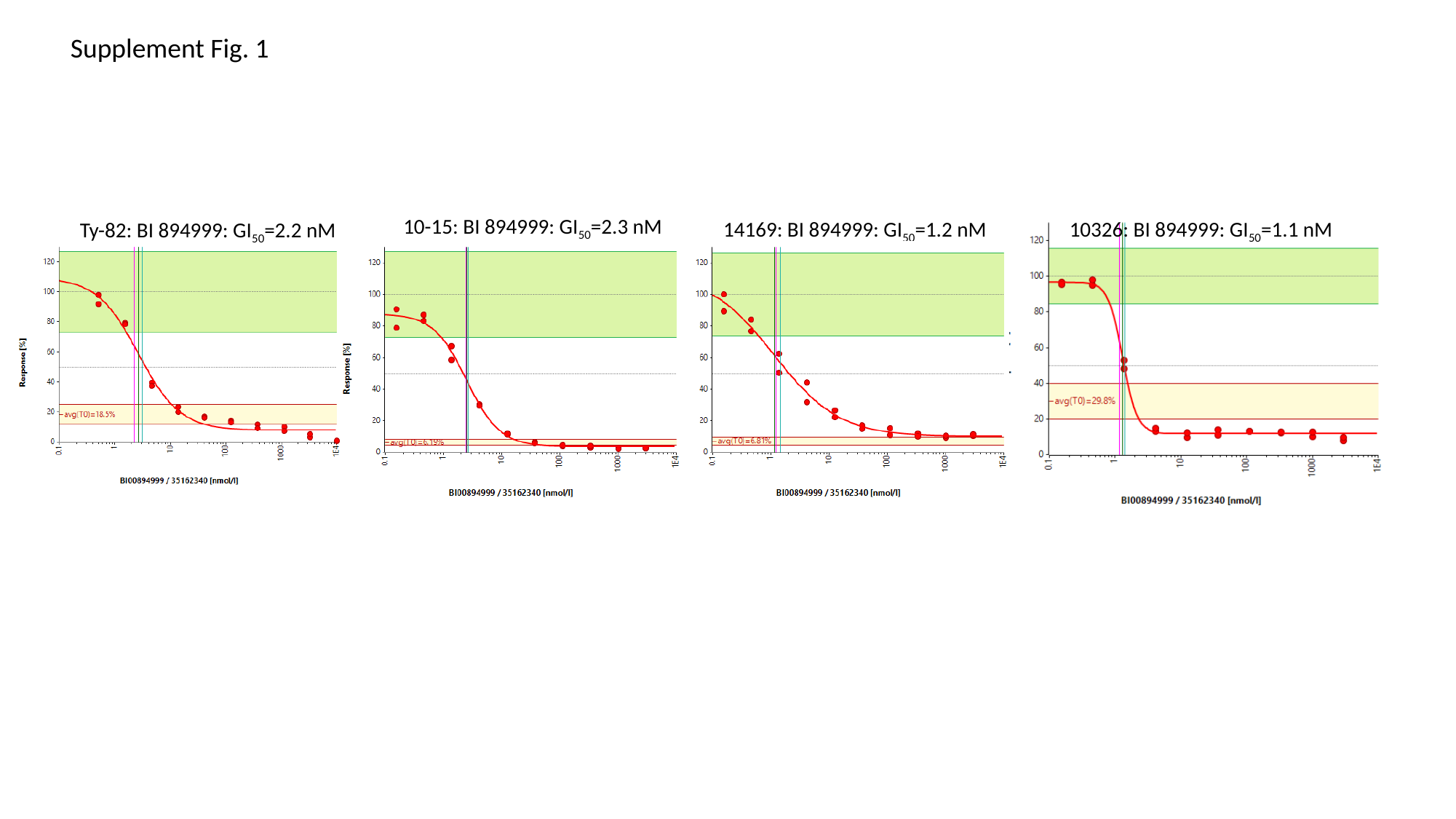

Supplement Fig. 1
10-15: BI 894999: GI50=2.3 nM
14169: BI 894999: GI50=1.2 nM
10326: BI 894999: GI50=1.1 nM
Ty-82: BI 894999: GI50=2.2 nM

## Slide 2
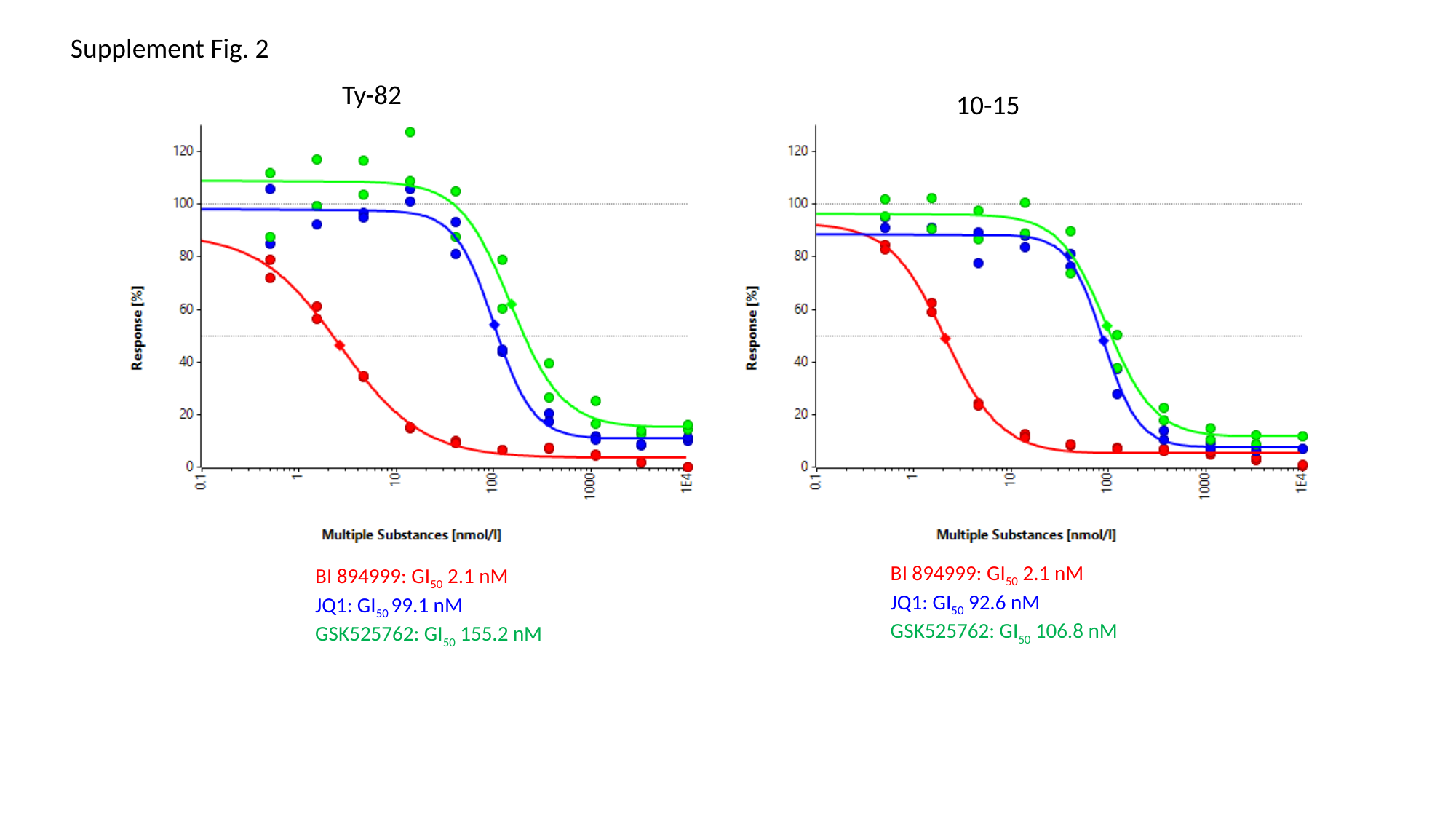

Supplement Fig. 2
Ty-82
10-15
BI 894999: GI50 2.1 nM
JQ1: GI50 92.6 nM
GSK525762: GI50 106.8 nM
BI 894999: GI50 2.1 nM
JQ1: GI50 99.1 nM
GSK525762: GI50 155.2 nM

## Slide 3
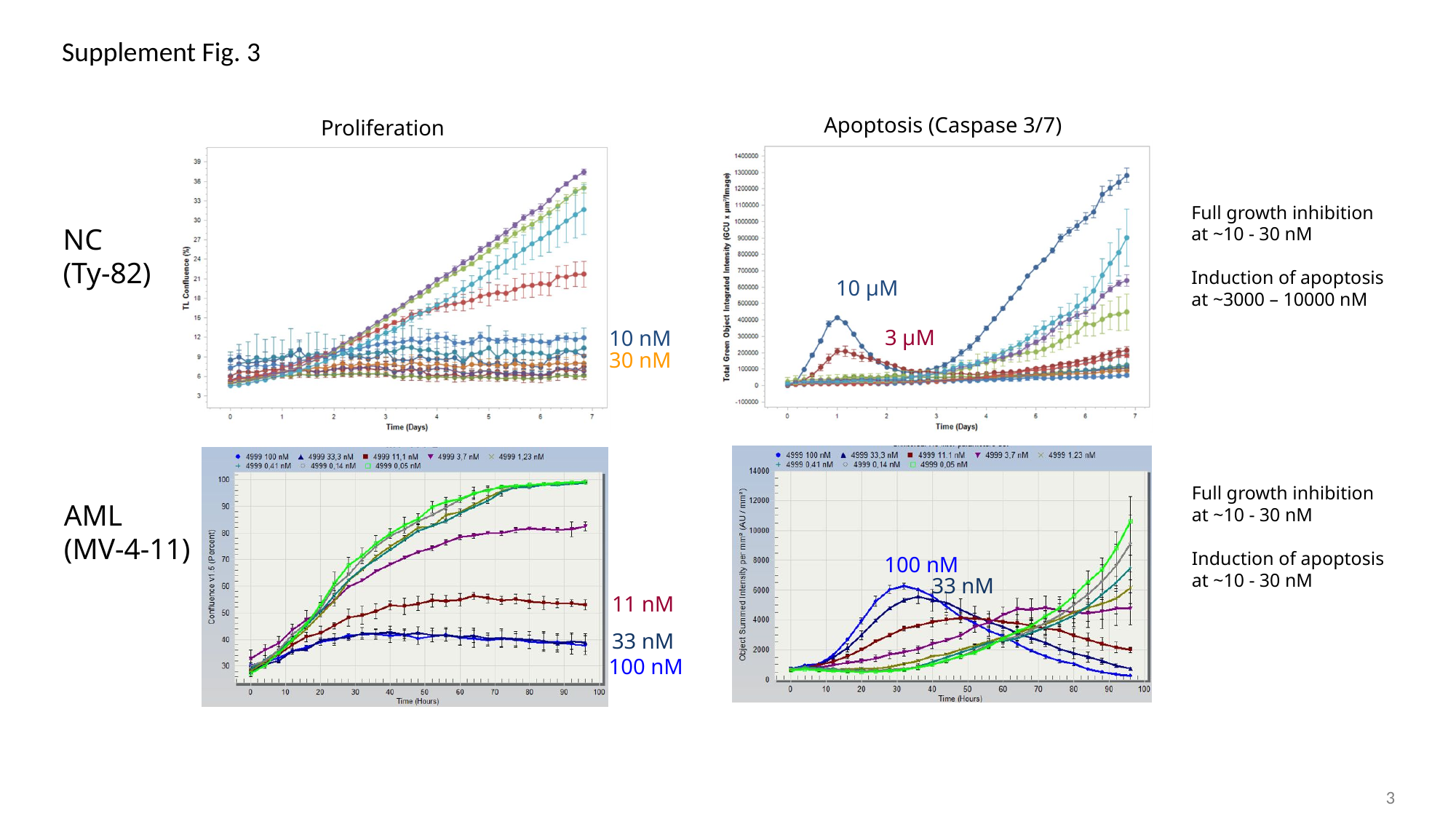

Supplement Fig. 3
Apoptosis (Caspase 3/7)
Proliferation
Full growth inhibition
at ~10 - 30 nM
Induction of apoptosis
at ~3000 – 10000 nM
NC
(Ty-82)
10 µM
3 µM
10 nM
30 nM
Full growth inhibition
at ~10 - 30 nM
Induction of apoptosis
at ~10 - 30 nM
AML
(MV-4-11)
100 nM
33 nM
11 nM
33 nM
100 nM
3

## Slide 4
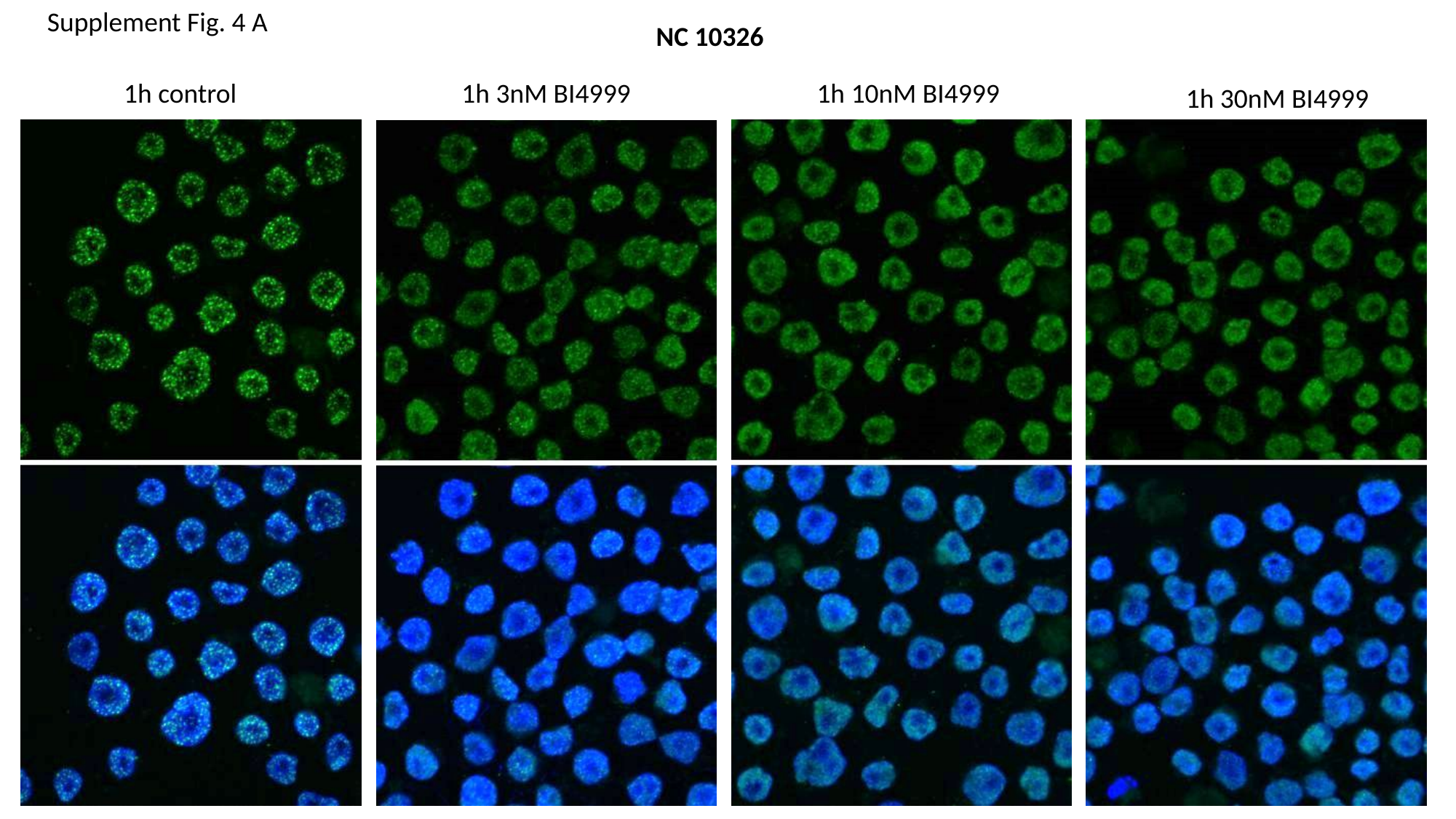

Supplement Fig. 4 A
NC 10326
1h control
1h 3nM BI4999
1h 10nM BI4999
1h 30nM BI4999

## Slide 5
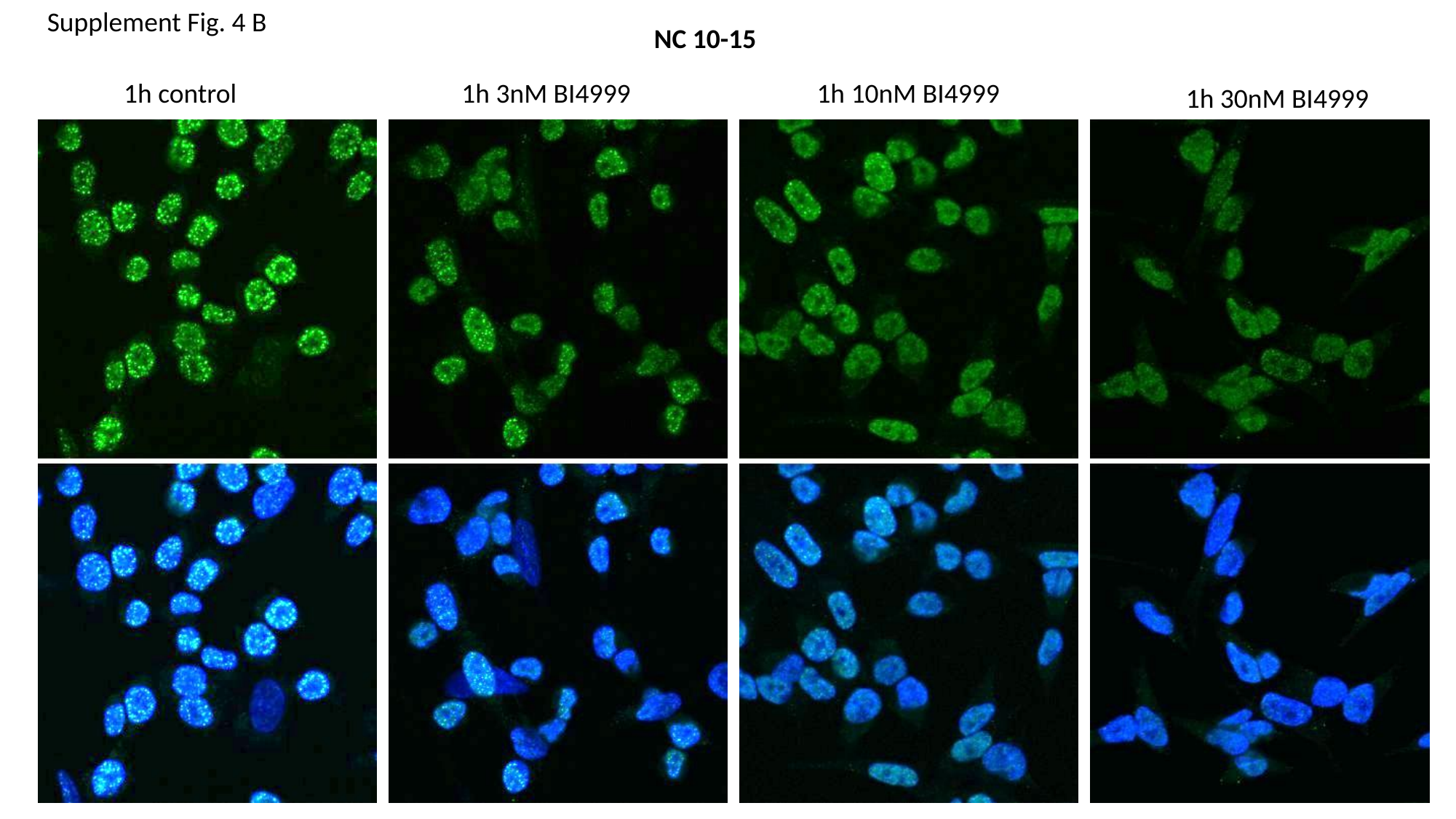

Supplement Fig. 4 B
NC 10-15
1h control
1h 3nM BI4999
1h 10nM BI4999
1h 30nM BI4999

## Slide 6
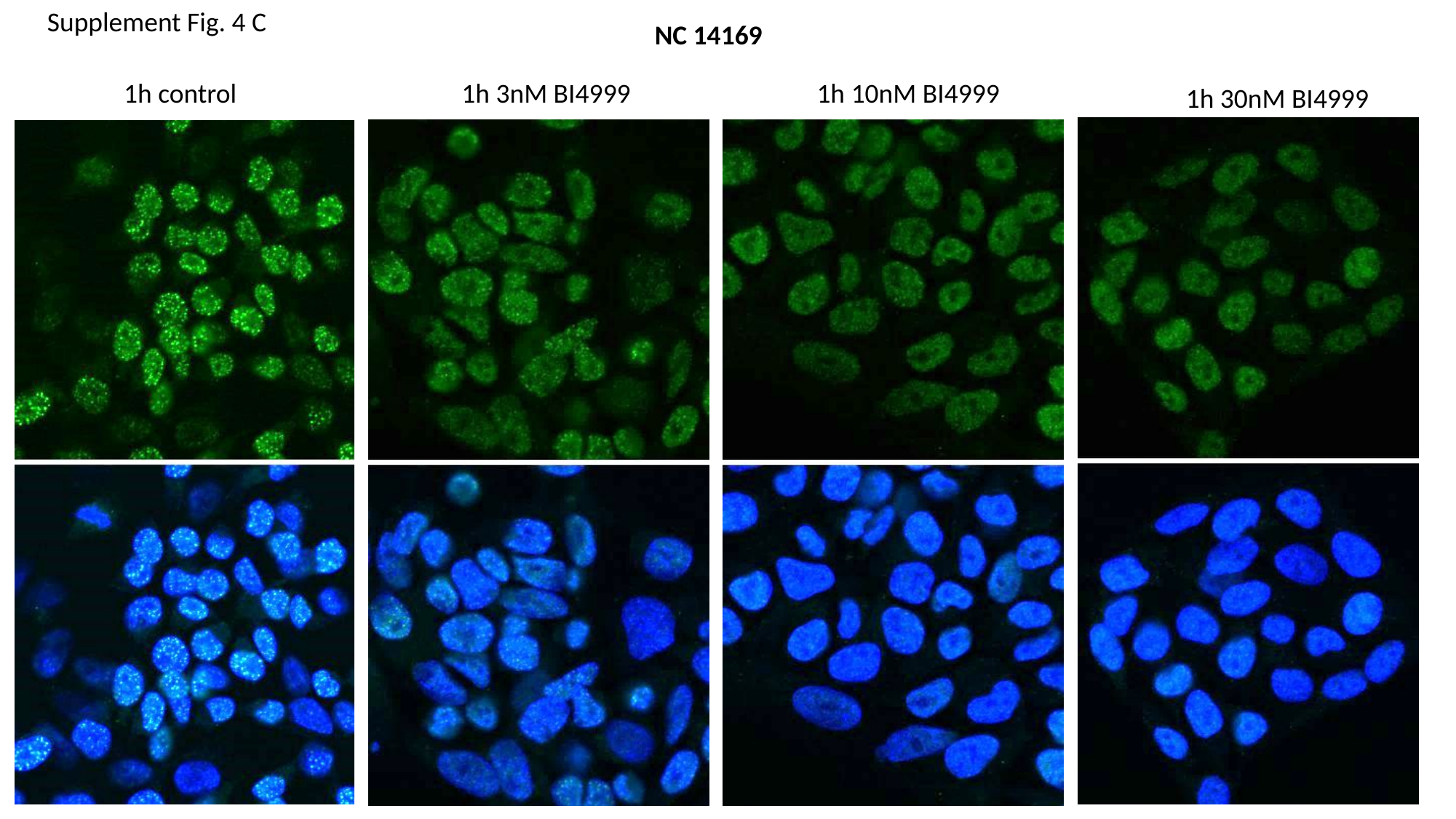

Supplement Fig. 4 C
NC 14169
1h control
1h 3nM BI4999
1h 10nM BI4999
1h 30nM BI4999

## Slide 7
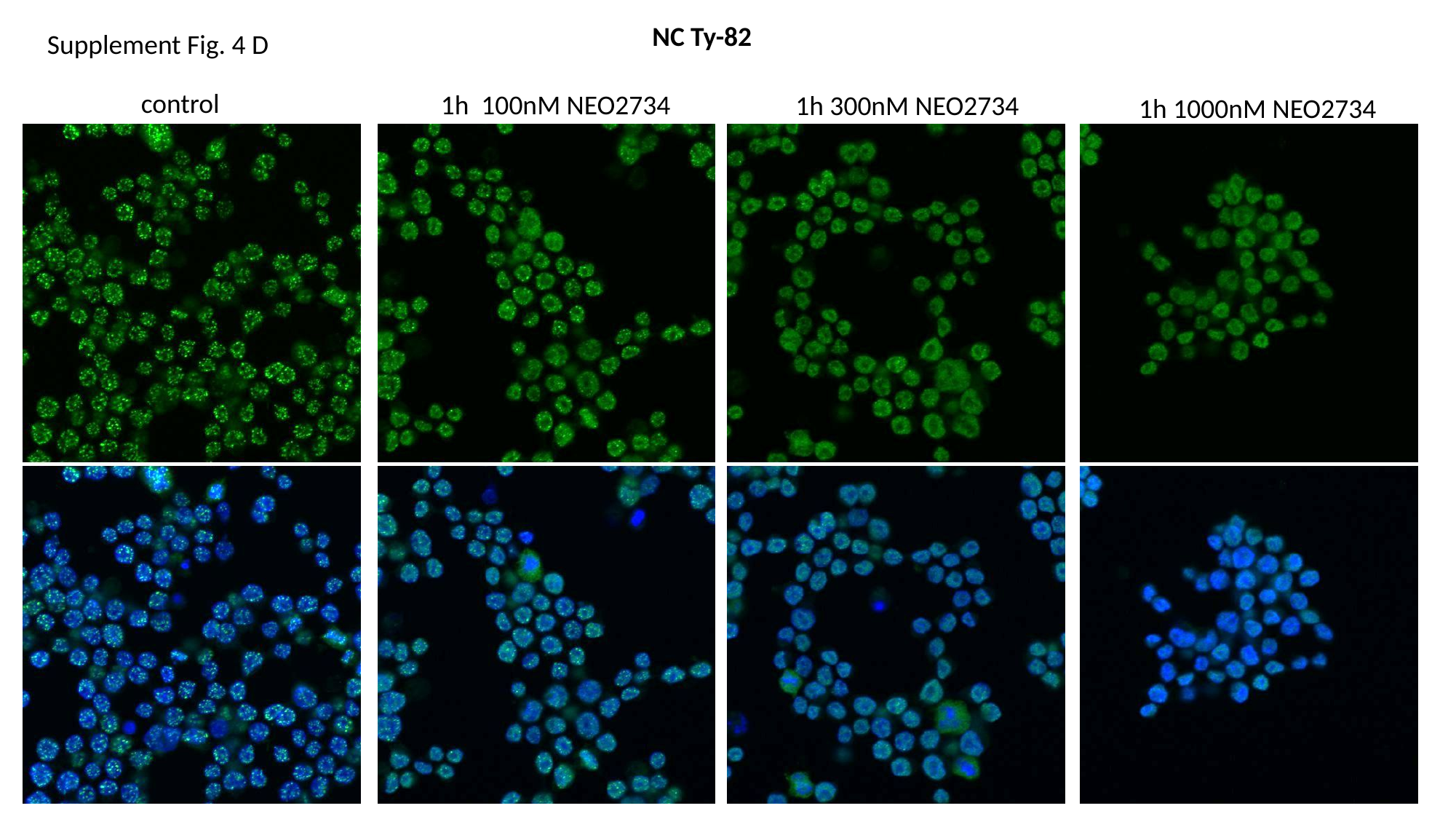

NC Ty-82
Supplement Fig. 4 D
control
1h 100nM NEO2734
1h 300nM NEO2734
1h 1000nM NEO2734

## Slide 8
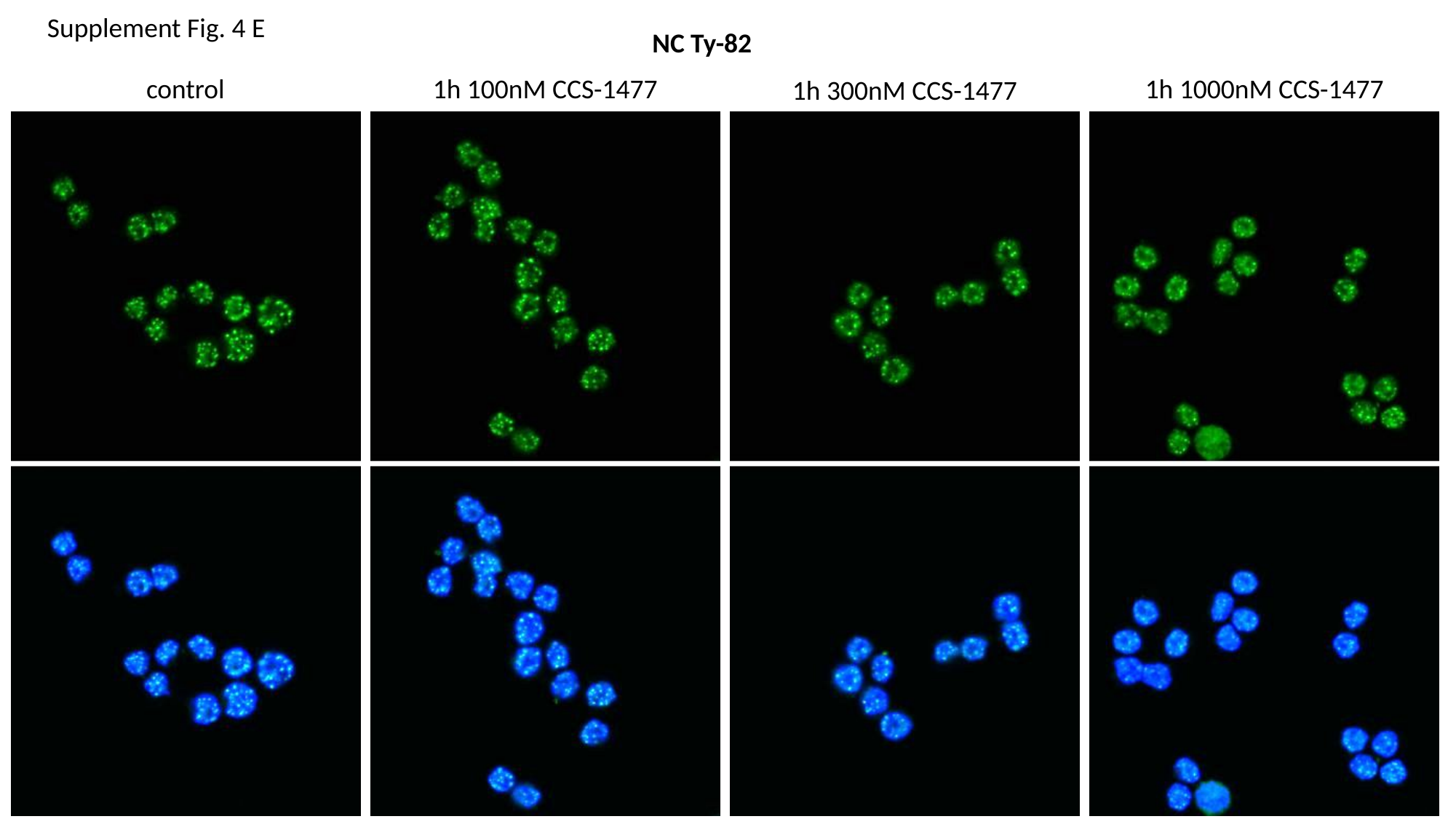

Supplement Fig. 4 E
NC Ty-82
1h 1000nM CCS-1477
control
1h 100nM CCS-1477
1h 300nM CCS-1477

## Slide 9
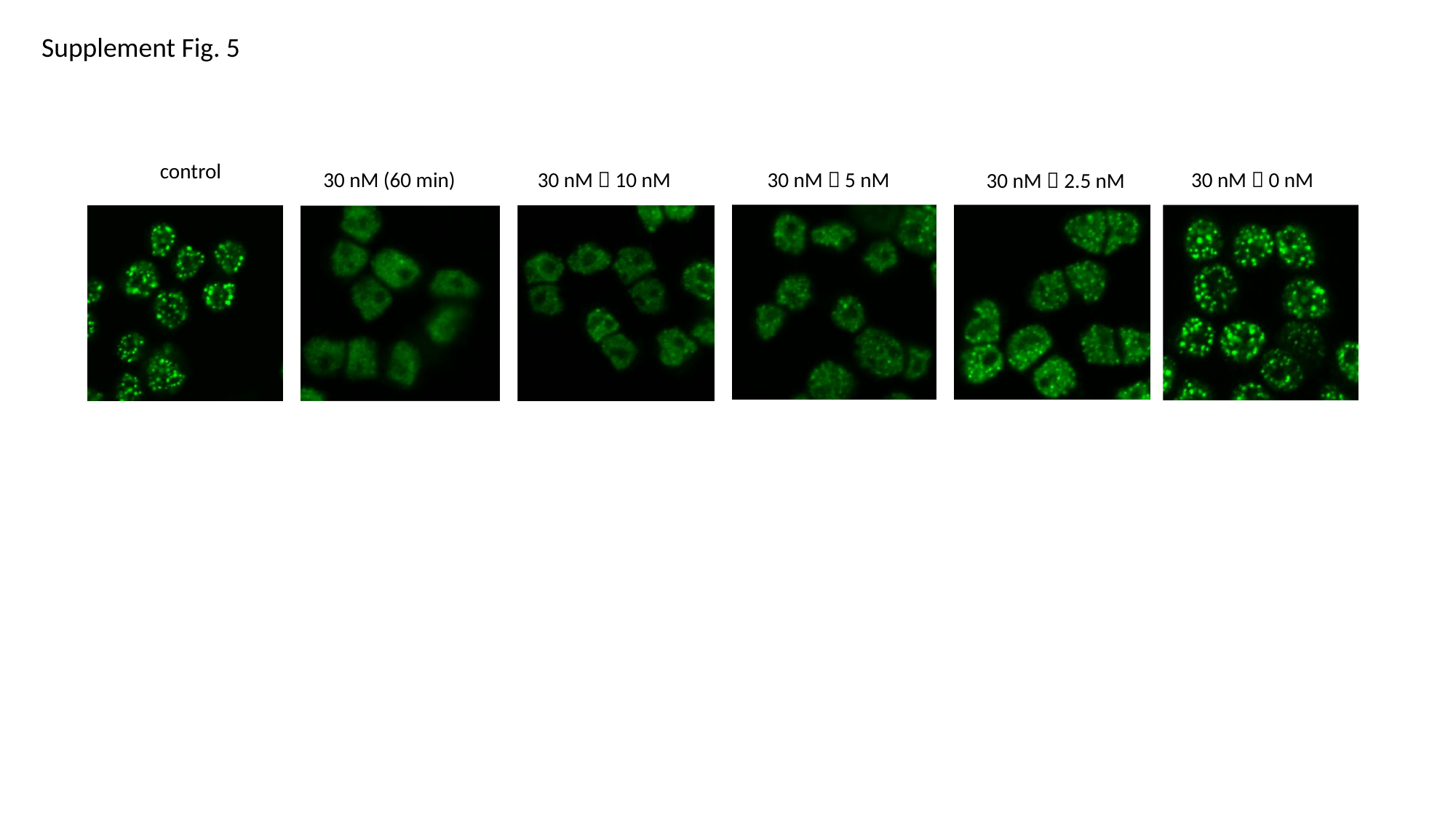

Supplement Fig. 5
control
30 nM  0 nM
30 nM (60 min)
30 nM  10 nM
30 nM  5 nM
30 nM  2.5 nM

## Slide 10
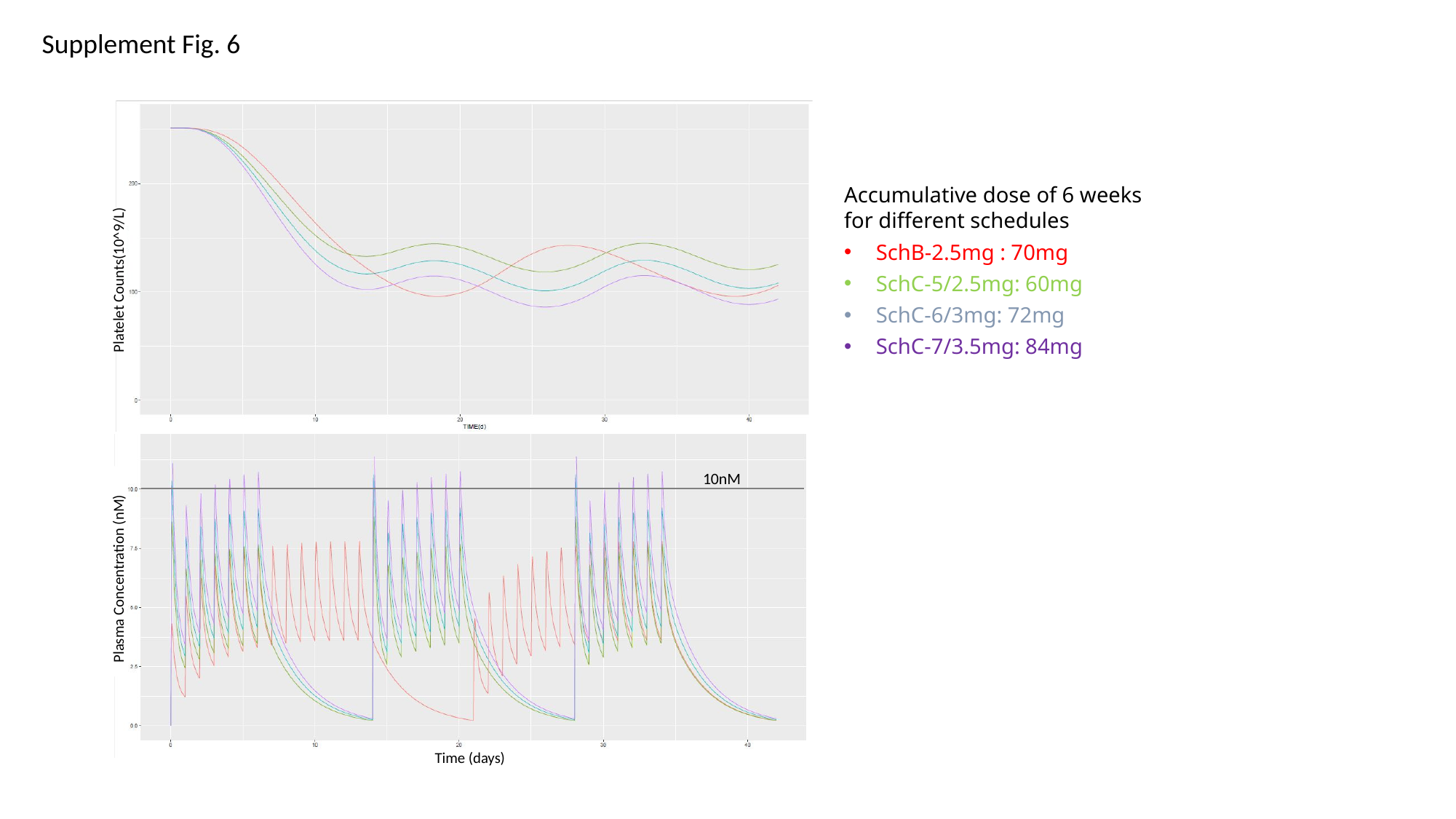

Supplement Fig. 6
Pharmacometrics modeling led to proposal of Schedule C
Accumulative dose of 6 weeks for different schedules
SchB-2.5mg : 70mg
SchC-5/2.5mg: 60mg
SchC-6/3mg: 72mg
SchC-7/3.5mg: 84mg
Platelet Counts(10^9/L)
 Plasma Concentration (nM)
10nM
Time (days)

## Slide 11
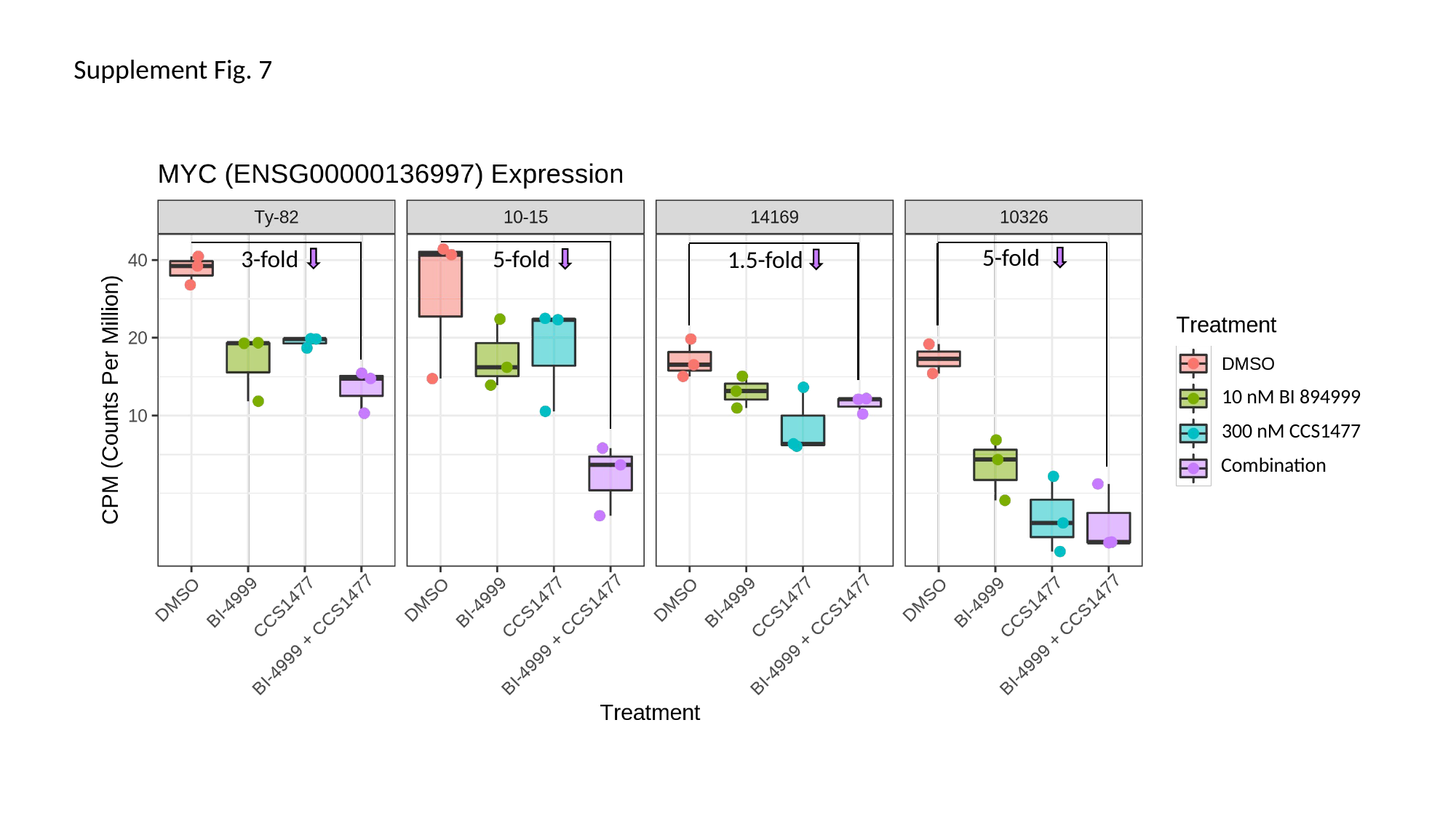

Supplement Fig. 7
5-fold
3-fold
5-fold
1.5-fold
10 nM BI 894999
300 nM CCS1477
Combination

## Slide 12
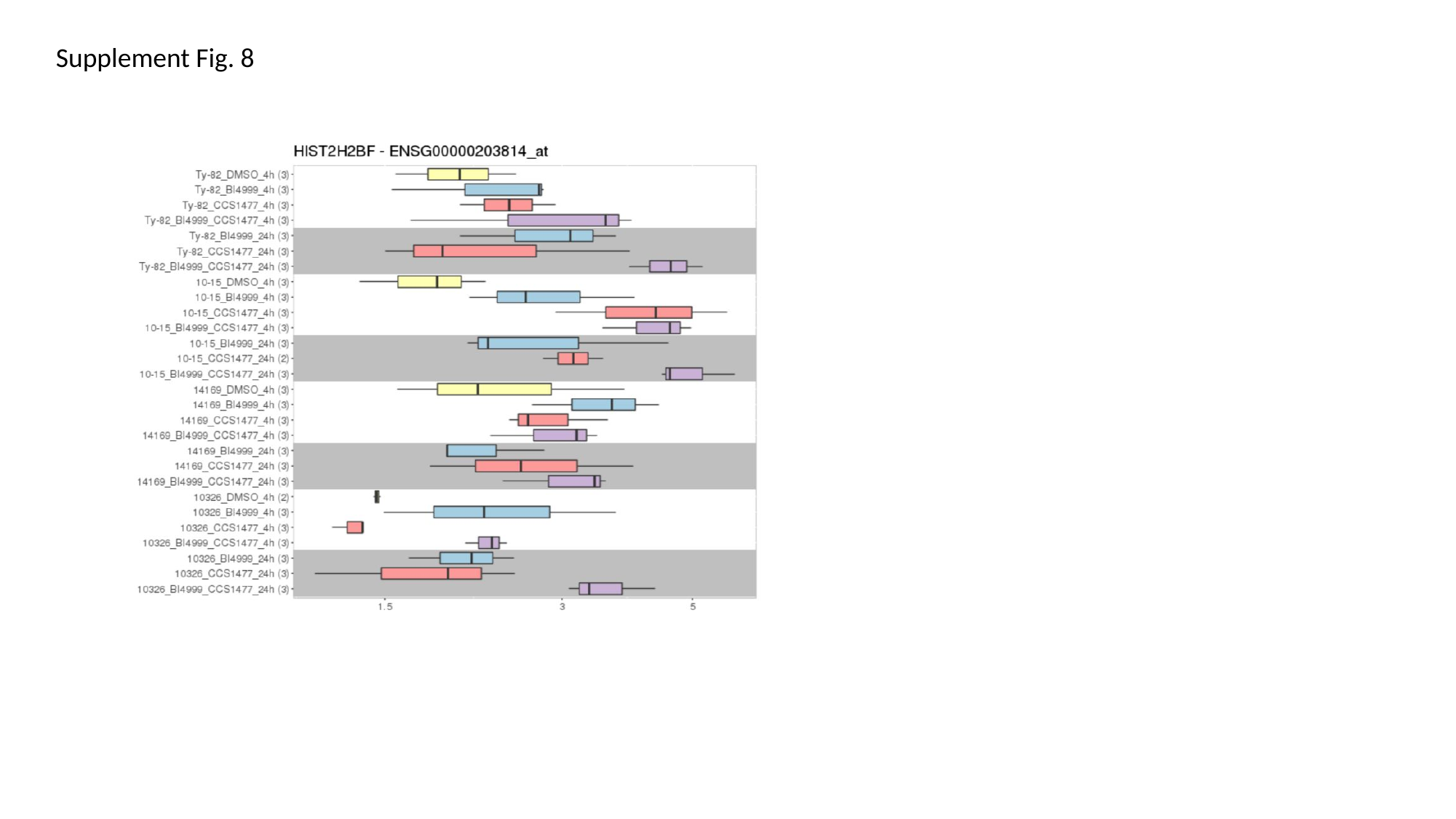

Supplement Fig. 8

## Slide 13
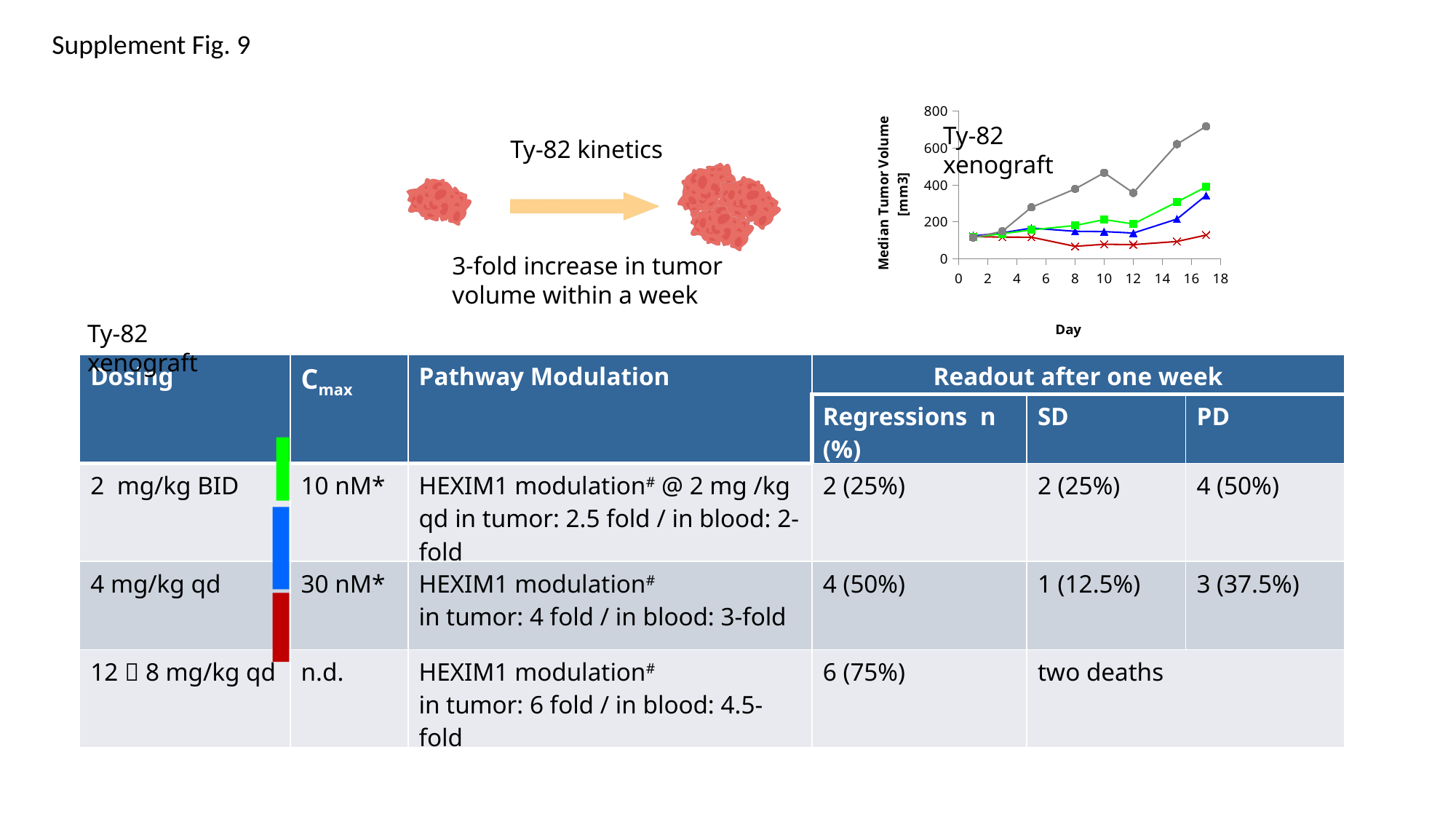

Supplement Fig. 9
### Chart
| Category | | | | |
|---|---|---|---|---|Ty-82 xenograft
Ty-82 kinetics
3-fold increase in tumor volume within a week
Ty-82 xenograft
| Dosing | Cmax | Pathway Modulation | Readout after one week | | |
| --- | --- | --- | --- | --- | --- |
| | | | Regressions n (%) | SD | PD |
| 2 mg/kg BID | 10 nM\* | HEXIM1 modulation# @ 2 mg /kg qd in tumor: 2.5 fold / in blood: 2-fold | 2 (25%) | 2 (25%) | 4 (50%) |
| 4 mg/kg qd | 30 nM\* | HEXIM1 modulation# in tumor: 4 fold / in blood: 3-fold | 4 (50%) | 1 (12.5%) | 3 (37.5%) |
| 12  8 mg/kg qd | n.d. | HEXIM1 modulation# in tumor: 6 fold / in blood: 4.5-fold | 6 (75%) | two deaths | |
 Readout after one week
13
*#Based on data from previous in vivo experiments in other model (NMRI-nude mice)

## Slide 14
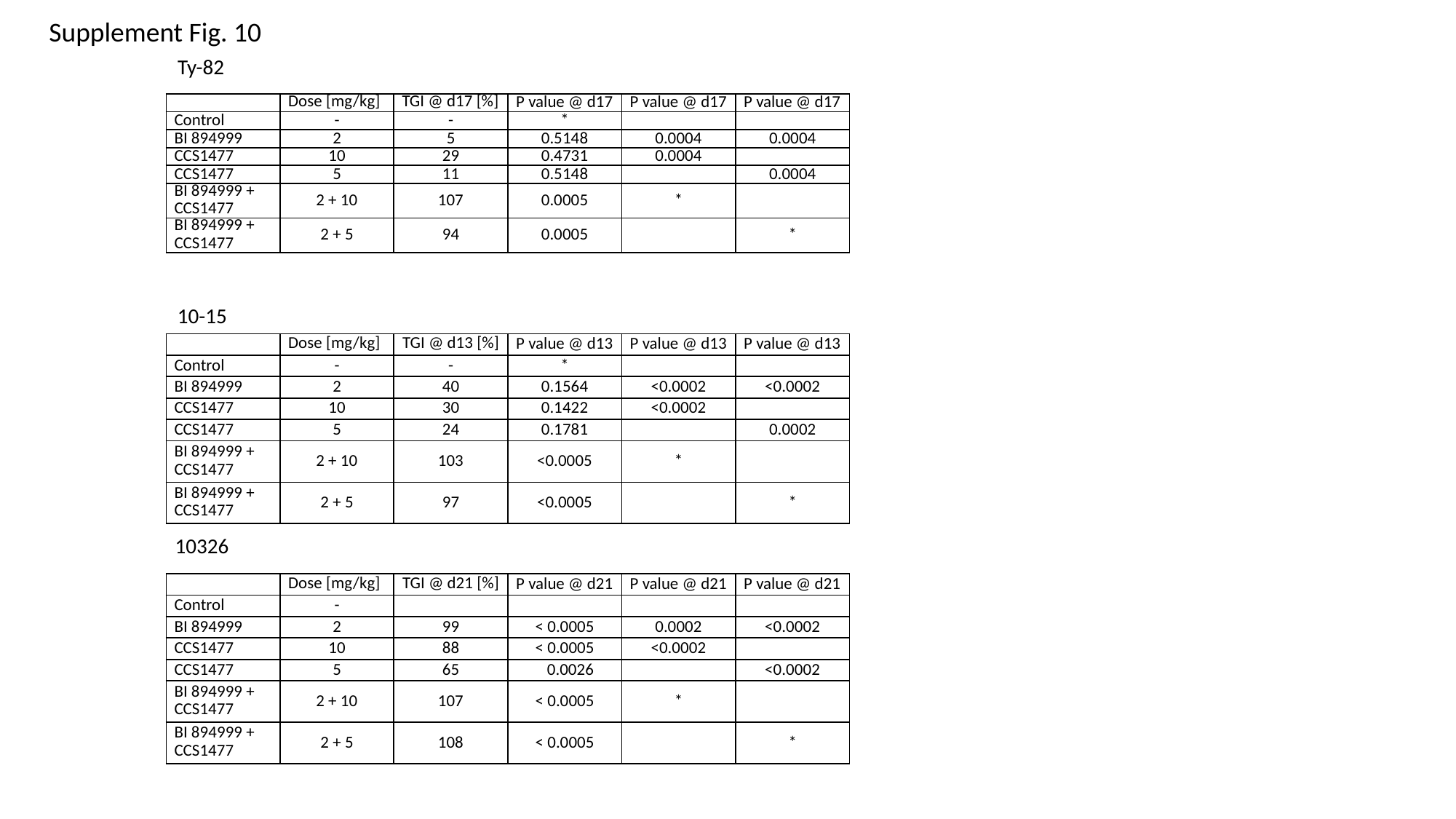

Supplement Fig. 10
Ty-82
| | Dose [mg/kg] | TGI @ d17 [%] | P value @ d17 | P value @ d17 | P value @ d17 |
| --- | --- | --- | --- | --- | --- |
| Control | - | - | \* | | |
| BI 894999 | 2 | 5 | 0.5148 | 0.0004 | 0.0004 |
| CCS1477 | 10 | 29 | 0.4731 | 0.0004 | |
| CCS1477 | 5 | 11 | 0.5148 | | 0.0004 |
| BI 894999 + CCS1477 | 2 + 10 | 107 | 0.0005 | \* | |
| BI 894999 + CCS1477 | 2 + 5 | 94 | 0.0005 | | \* |
10-15
| | Dose [mg/kg] | TGI @ d13 [%] | P value @ d13 | P value @ d13 | P value @ d13 |
| --- | --- | --- | --- | --- | --- |
| Control | - | - | \* | | |
| BI 894999 | 2 | 40 | 0.1564 | <0.0002 | <0.0002 |
| CCS1477 | 10 | 30 | 0.1422 | <0.0002 | |
| CCS1477 | 5 | 24 | 0.1781 | | 0.0002 |
| BI 894999 + CCS1477 | 2 + 10 | 103 | <0.0005 | \* | |
| BI 894999 + CCS1477 | 2 + 5 | 97 | <0.0005 | | \* |
10326
| | Dose [mg/kg] | TGI @ d21 [%] | P value @ d21 | P value @ d21 | P value @ d21 |
| --- | --- | --- | --- | --- | --- |
| Control | - | - | \* | | |
| BI 894999 | 2 | 99 | < 0.0005 | 0.0002 | <0.0002 |
| CCS1477 | 10 | 88 | < 0.0005 | <0.0002 | |
| CCS1477 | 5 | 65 | 0.0026 | | <0.0002 |
| BI 894999 + CCS1477 | 2 + 10 | 107 | < 0.0005 | \* | |
| BI 894999 + CCS1477 | 2 + 5 | 108 | < 0.0005 | | \* |

## Slide 15
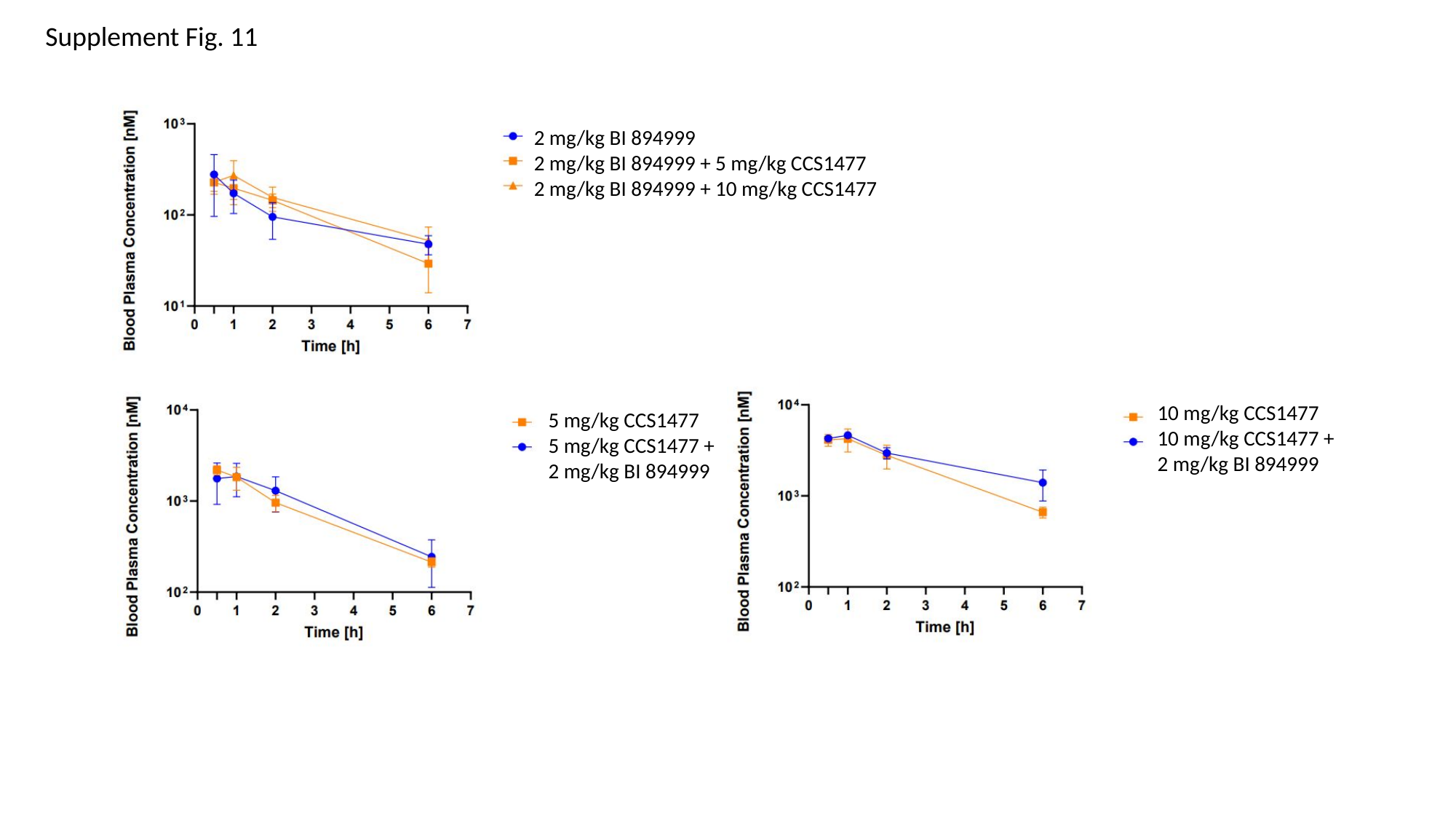

Supplement Fig. 11
2 mg/kg BI 894999
2 mg/kg BI 894999 + 5 mg/kg CCS1477
2 mg/kg BI 894999 + 10 mg/kg CCS1477
10 mg/kg CCS1477
10 mg/kg CCS1477 +
2 mg/kg BI 894999
5 mg/kg CCS1477
5 mg/kg CCS1477 +
2 mg/kg BI 894999

## Slide 16
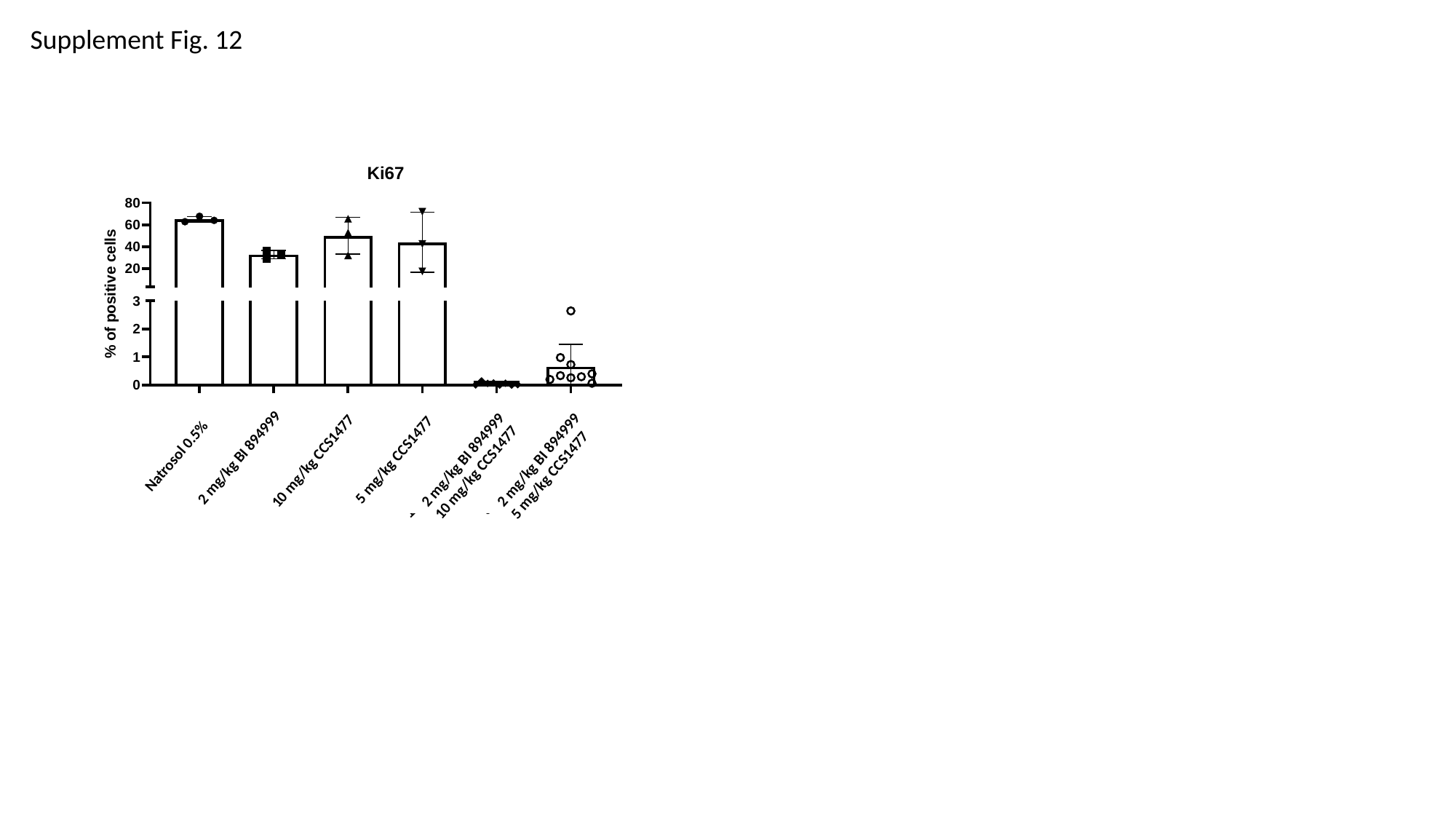

Supplement Fig. 12
Natrosol 0.5%
2 mg/kg BI 894999
5 mg/kg CCS1477
2 mg/kg BI 894999
10 mg/kg CCS1477
2 mg/kg BI 894999
5 mg/kg CCS1477
10 mg/kg CCS1477

## Slide 17
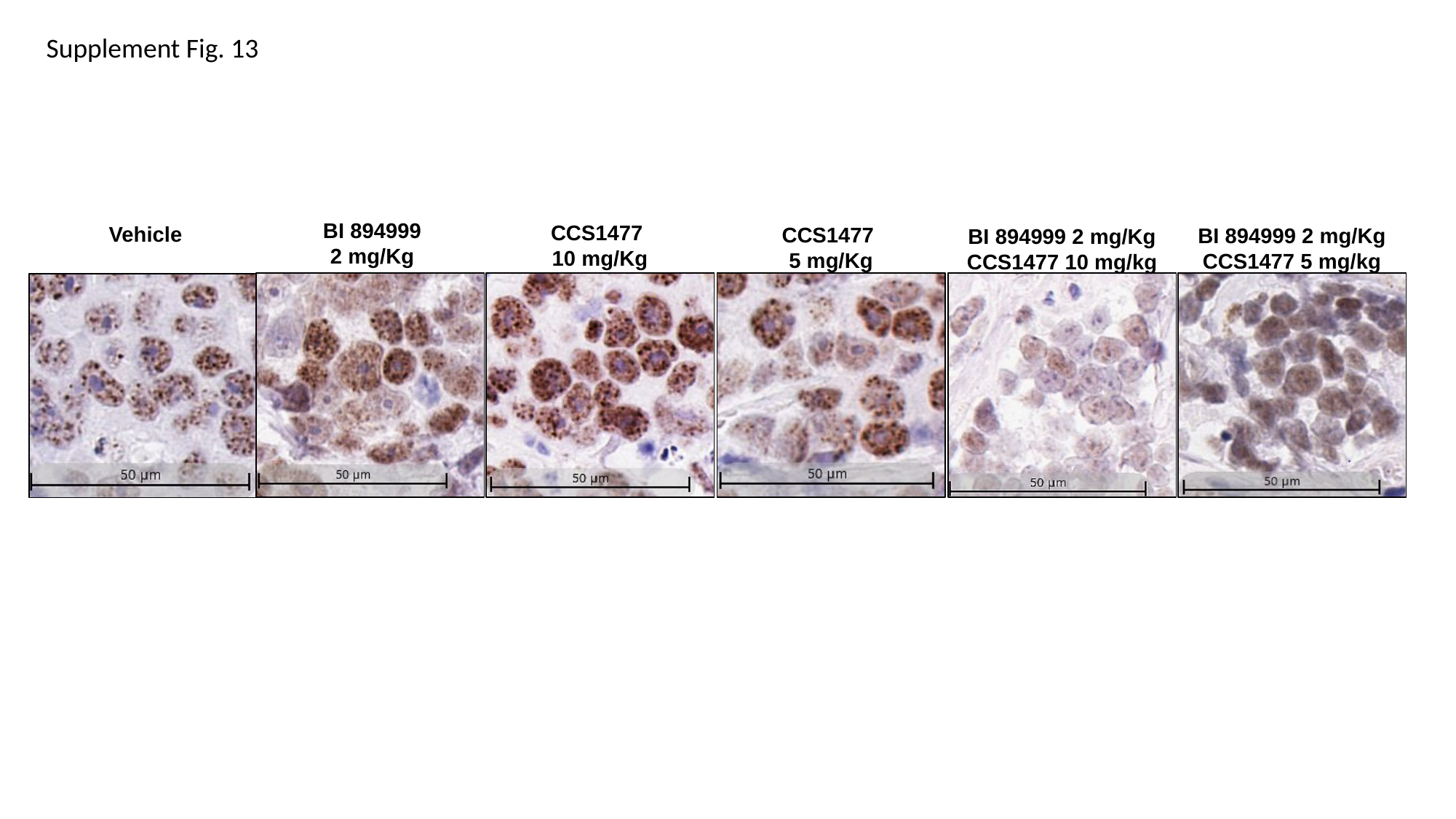

Supplement Fig. 13
BI 894999
2 mg/Kg
CCS1477
10 mg/Kg
Vehicle
CCS1477
5 mg/Kg
BI 894999 2 mg/Kg
CCS1477 5 mg/kg
BI 894999 2 mg/Kg
CCS1477 10 mg/kg

## Slide 18
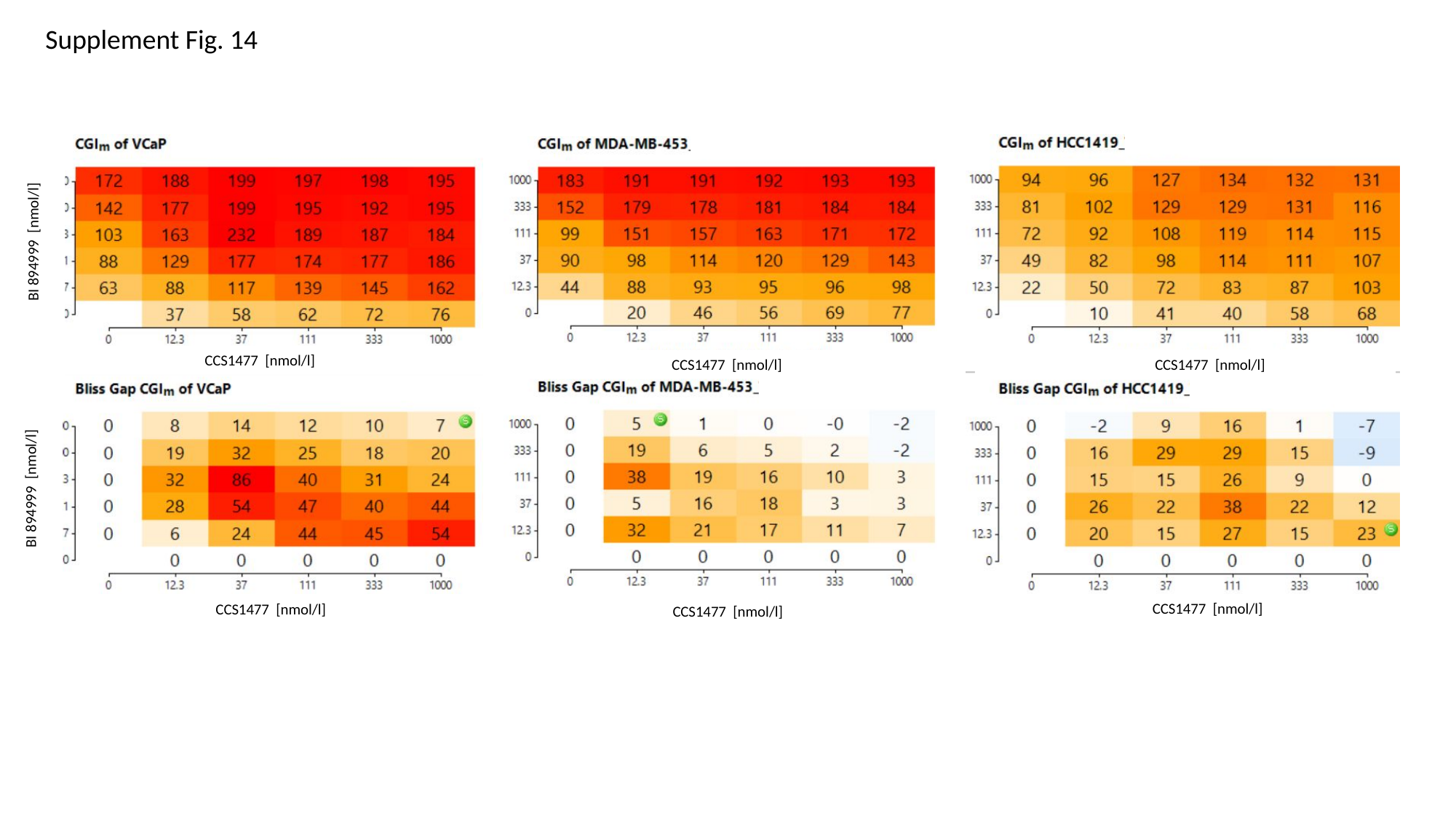

Supplement Fig. 14
 BI 894999 [nmol/l]
 CCS1477 [nmol/l]
 CCS1477 [nmol/l]
 CCS1477 [nmol/l]
 BI 894999 [nmol/l]
 CCS1477 [nmol/l]
 CCS1477 [nmol/l]
 CCS1477 [nmol/l]
