# Supplementary material for: Therapeutic impact of BET inhibitor BI 894999 treatment: backtranslation from the clinic
Source: Br J Cancer. 2022 Apr 20;127(3):577–86. doi: 10.1038/s41416-022-01815-5 (PMC9346113; doi:10.1038/s41416-022-01815-5)
Supplement: Supplementary file 2 — Figure Legend Supplement [file 41416_2022_1815_MOESM2_ESM.docx]

Figure Legends Supplement

Supplement Fig. 1

Dose response curves (96h cell proliferation assay - Alamar readout) on four NC cell lines with BET inhibitor BI 894999. Yellow bar: readout t zero (before adding of compound). Green bar: DMSO control (max proliferation).

Supplement Fig. 2

Dose response curves (96h cell proliferation assay - Alamar readout) on four NC cell lines with multiple BET inhibitors.

Supplement Fig. 3

Incucyte analysis. Ty-82 cells (NC) or MV-4-11 (AML) cells were treated with BI 894999 at the concentrations indicated and analyzed over time with the IncuCyte™Caspase-3/7 Apoptosis Assay Reagent (Essenbioscience Cat No 4440). Cell confluence (left) and green fluorescence as a measure of caspase 3/7 activation (right) were monitored for up to 7 days with images taken every 3 hours.

Supplement Fig. 4 (A-E)

Immunofluorescence staining of NUT megadomains on the NC cell lines treated with BI 894999 (A-C) or NEO2734 (D) or CCS1477 (E) for 1 hour at the indicated concentrations. Upper row: antibody against NUT, lower row: DAPI.

Supplement Fig. 5

Immunofluorescence staining of NUT megadomains on NC Ty-82 treated with BI with BI 894999 30nM for 1 h, then changed to 10 nM or 5 nM or 2.5 nM or control for 24 h.

Supplement Fig. 6

Pharmacometrics simulation for platelet counts (top) and plasma concentration (bottom) as a function of four different dosing regimens of BI 894999. Solid line of 10nM indicated the estimated plasma concentration needed to stimulate a maximum 2-fold change from baseline for HEXIM1.

Supplement Fig. 7

Modulation of PD marker MYC. Expression analysis by RNAseq in 4 NC cell lines. Cells were treated for 4 h with DMSO control, 10 nM BI 894999, 300 nM CCS1477 or a combination of both. Expression values are indicated in CPM (counts per million). Increased suppression of MYC upon combination.

Supplement Fig. 8

Modulation of PD marker HIST2H2BF. Expression analysis by RNAseq in 4 NC cell lines. Cells were treated for 4 h with DMSO control, 10 nM BI 894999, 300 nM CCS1477 or a combination of both. Expression values are indicated in CPM (counts per million). Very low baseline expression of HIST2H2BF.

Supplement Fig. 9

C_max_ and PD modulation of Ty-82 tumors treated with BI 894999. 4 mg/kg in Ty-82 NC are comparable to Sch C (6mg/3mg) in humans.

Supplement Fig. 10

*In vivo* combination of BI 894999 with CCS1477.

One-sided non-parametric Mann-Whitney-Wilcoxon U-tests were applied to compare all treatment groups with the control, as well as the combination therapy group with the corresponding monotherapy groups. p values were adjusted for multiple comparisons according to Bonferroni-Holm.

Supplement Fig. 11

PK analysis in mouse plasma. Oral treatment at the indicated doses. Sampling at the indicated timepoints.

Supplement Fig. 12

Quantification of Ki67 staining in NC 10326 tumor sections after treatment with BI 894999, CCS1477, or the combination thereof.

Supplement Fig. 13

Histopathological analysis.

BRD3-NUT 10326 tumor sections after treatment with 2 mg/kg BI 894999, 5 mg/kg or 10 mg/kg CCS1477 and the combination of both. IHC staining for NUT protein.

Loss of speckled staining in the combinations.

Supplement Fig. 14

*In vitro* combination of BI 894999 and CCS1477 in prostate cancer cell line VCaP and breast cancer cell lines MDA-MB-453 and HCC1419.

BI 894999 combined with CCS1477 and analyzed for Bliss synergy. Combination platemap in a 6x6 matrix (duplicates for each setting) at the indicated concentrations. CGI (cell growth inhibition) Values <100%: tumor cell growth, CGI 100%: tumor cell stasis and GCI>100%: tumor cell killing.
